# Distinct molecular profile of diffuse cerebellar gliomas

**DOI:** 10.1007/s00401-017-1771-1

**Published:** 2017-08-29

**Authors:** Masashi Nomura, Akitake Mukasa, Genta Nagae, Shogo Yamamoto, Kenji Tatsuno, Hiroki Ueda, Shiro Fukuda, Takayoshi Umeda, Tomonari Suzuki, Ryohei Otani, Keiichi Kobayashi, Takashi Maruyama, Shota Tanaka, Shunsaku Takayanagi, Takahide Nejo, Satoshi Takahashi, Koichi Ichimura, Taishi Nakamura, Yoshihiro Muragaki, Yoshitaka Narita, Motoo Nagane, Keisuke Ueki, Ryo Nishikawa, Junji Shibahara, Hiroyuki Aburatani, Nobuhito Saito

**Affiliations:** 1 Department of Neurosurgery, Graduate School of Medicine, The University of Tokyo, 7-3-1 Hongo, Bunkyo-ku, Tokyo, 113-8655 Japan; 2 Genome Science Division, Research Center for Advanced Science and Technology (RCAST), The University of Tokyo, 4-6-1 Komaba, Meguro-ku, Tokyo, 153-8904 Japan; 3 Department of Neuro-Oncology/Neurosurgery, Saitama International Medical Center, Saitama Medical University, 1397-1 Yamane, Hidaka-shi, Saitama 350-1298 Japan; 4 Department of Neurosurgery, Dokkyo Medical University, 880 Kitakobayashi, Mibu-machi, Shimotsuga-gun, Tochigi 321-0293 Japan; 5 Department of Neurosurgery, Kyorin University Faculty of Medicine, 6-20-2 Shinkawa, Mitaka, Tokyo 181-8611 Japan; 6 Department of Neurosurgery, Tokyo Women’s Medical University, 8-1, Kawada-cho, Shinjuku-ku, Tokyo, 162-8666 Japan; 7 Division of Brain Tumor Translational Research, National Cancer Center Research Institute, 5-1-1 Tsukiji, Chuo-ku, Tokyo, 104-0045 Japan; 8 Department of Neurosurgery, Graduate School of Medicine, Yokohama City University, 3-9, Fukuura, Kanazawa-ku, Yokohama, 236-0004 Japan; 9 Department of Neurosurgery and Neuro-Oncology, National Cancer Center Hospital, 5-1-1 Tsukiji, Chuo-ku, Tokyo, 104-0045 Japan; 10 Department of Pathology, Kyorin University Faculty of Medicine, 6-20-2 Shinkawa, Mitaka, Tokyo 181-8611 Japan

**Keywords:** Glioma, Cerebellum, Genomics, Gene expression, DNA methylation

## Abstract

Recent studies have demonstrated that tumor-driving alterations are often different among gliomas that originated from different brain regions and have underscored the importance of analyzing molecular characteristics of gliomas stratified by brain region. Therefore, to elucidate molecular characteristics of diffuse cerebellar gliomas (DCGs), 27 adult, mostly glioblastoma cases were analyzed. Comprehensive analysis using whole-exome sequencing, RNA sequencing, and Infinium methylation array (*n* = 17) demonstrated their distinct molecular profile compared to gliomas in other brain regions. Frequent mutations in chromatin-modifier genes were identified including, noticeably, a truncating mutation in *SETD2* (*n* = 4), which resulted in loss of H3K36 trimethylation and was mutually exclusive with *H3F3A* K27M mutation (*n* = 3), suggesting that epigenetic dysregulation may lead to DCG tumorigenesis. Alterations that cause loss of p53 function including *TP53* mutation (*n* = 9), *PPM1D* mutation (*n* = 2), and a novel type of *PPM1D* fusion (*n* = 1), were also frequent. On the other hand, mutations and copy number changes commonly observed in cerebral gliomas were infrequent. DNA methylation profile analysis demonstrated that all DCGs except for those with *H3F3A* mutations were categorized in the “RTK I (PDGFRA)” group, and those DCGs had a gene expression signature that was highly associated with *PDGFRA*. Furthermore, compared with the data of 315 gliomas derived from different brain regions, promoter methylation of transcription factors genes associated with glial development showed a characteristic pattern presumably reflecting their tumor origin. Notably, *SOX10*, a key transcription factor associated with oligodendroglial differentiation and *PDGFRA* regulation, was up-regulated in both DCG and H3 K27M-mutant diffuse midline glioma, suggesting their developmental and biological commonality. In contrast, *SOX10* was silenced by promoter methylation in most cerebral gliomas. These findings may suggest potential tailored targeted therapy for gliomas according to their brain region, in addition to providing molecular clues to identify the region-related cellular origin of DCGs.

**Electronic supplementary material:**

The online version of this article (doi:10.1007/s00401-017-1771-1) contains supplementary material, which is available to authorized users.

## Introduction

Diffuse glioma in the cerebellum is infrequent, accounting for 0.6–3.3% of all gliomas [1, 10, 18]. Previous studies reported that patients with diffuse cerebellar glioma (DCG) are younger in general, and that DCGs have a relatively smaller tumor volume compared to cerebral gliomas [1, 18].

Recent comprehensive genetic analysis of gliomas demonstrated that common alterations that contribute to tumorigenesis differ according to the original tumor region in the central nervous system as well as with the patient’s age [47]. For example, a K27M mutation in *H3F3A*, which encodes the replication-independent histone 3 variant H3.3, is predominantly found in pediatric and young adult high-grade gliomas located in a midline structure such as the brainstem, thalamus, or spinal cord, whereas the G34R/V mutation is associated with adolescent glioblastoma (GBM) in cerebral hemispheres [2, 16, 43, 48]. Ependymoma, a different histological type of glioma, was also demonstrated to have a different molecular profile according to the anatomical region of the original tumor; oncogenic fusions involving *RELA* or *YAP1* were generally seen in supratentorial ependymomas, whereas posterior fossa ependymomas had an extremely low number of mutations, and their pediatric subset showed a typical DNA methylation pattern [26, 31, 32]. Importantly, tumors of different molecular backgrounds show different responses to therapy, leading to different prognoses. Furthermore, identification of tumor-driving molecular alterations in each case would allow selection of relevant molecular targeting drugs that may become available through extensive research in the era of precision medicine. Thus, it is of increasing importance to clarify the molecular background of tumors that may have specific biological traits. However, DCGs, which may be biologically different from common types of gliomas such as those located in cerebral hemispheres, have not been well characterized molecularly, partly due to their relative rarity. As a consequence, it is still unclear whether the diagnostic or therapeutic approaches for cerebral gliomas are applicable to cerebellar gliomas.

To determine the characteristics of cerebellar glioma, we here performed comprehensive molecular profiling of these gliomas including whole-exome sequencing (WES), Infinium methylation array, and RNA sequencing and compared their profile with that of gliomas derived from other anatomical regions. We demonstrated that DCGs have a region-related characteristic molecular profile that may shed light on the cellular origin of DCG, and also could be specifically targeted as a future treatment strategy.

## Materials and methods

### Clinical samples

Clinical samples were obtained from individuals who underwent surgery at The University of Tokyo Hospital, Kyorin University Hospital, Dokkyo Medical University Hospital, Saitama Medical University International Medical Center, Tokyo Women’s Medical University Hospital, Yokohama City University Hospital, and the National Cancer Center Hospital, with the patient’s informed consent. This study was approved by the ethics committees of each institute.

We only used samples that were radiographically confined to the cerebellum, and cases that had multiple lesions located outside of the cerebellum or had a tumor extending to the brainstem were excluded (Online Resource 1: Fig. S1). Samples were histologically diagnosed according to 2016 World Health Organization (WHO) classification by an experienced neuropathologist in each hospital and were further reviewed by a senior neuropathologist (J.S.) [25]. Of the 27 DCGs available in this study, 22 were freshly frozen tumors and five were formalin-fixed paraffin-embedded (FFPE) tissues. Of the 22 freshly frozen tumors, matched normal blood was obtained in 17 cases. Only these 17 samples could be analyzed by WES and methylation array, and such comprehensive analyses were not possible for the other 10 cases, because only a small amount or low-quality DNA was obtained from these remaining cases.

For comparison of the gene expression profile, eight cerebral GBM samples were also analyzed. Detailed information of the samples used in this study is provided in Online Resource 2: Table S1.

### DNA and RNA extraction

The DNeasy Blood and Tissue kit (Qiagen) was used to extract DNA from tumor tissue and paired normal blood according to the manufacturer’s instructions. The RNeasy Mini kit (Qiagen) was used to extract RNA from freshly frozen tumor tissue. The Qubit fluorometer (Life Technologies) was used to measure the concentration of double-stranded DNA. The Tape station (Agilent Technologies) was used to measure the quality of RNA.

### Sanger sequencing

Sanger sequencing was performed to detect the hotspot mutation of *IDH1* (R132), *IDH2* (R172), *TERT* promoter (C228 and C250), and *H3F3A* (K27). The oligo primers used for PCR amplification of these genes and the annealing temperature for each primer set are shown in Online Resource 2: Table S2. The high-fidelity DNA polymerase KOD-Plus-Neo (Toyobo) was used for PCR, and optimized thermal conditions were used. For each primer set, the PCR amplicon was gel-purified and then sequenced. Sanger sequencing was also performed for validation of mutations identified by WES.

### Immunohistochemistry

Immunohistochemical analysis was performed with 4-μm-thick FFPE tumor tissue sections. Briefly, after deparaffinization, antigen retrieval was performed for 30 min in citrate buffer (pH 6.0). The slides were then incubated with the following primary antibodies: H3 K27M (Millipore, ABE419, 1:500), H3K36 trimethylation (Abcam, ab9050, 1:2000).

### WES

WES was performed for 17 DCGs and matched blood samples (Online Resource 2: Table S1) as previously described [3, 20, 51]. In brief, DNA was fragmented using the Covaris SS Ultrasonicator. Exome capture was performed with Agilent SureSelect V6 plus COSMIC (Agilent Technologies). Each sample was sequenced with the HiSeq 2000 (Illumina) as 100-bp pair-ended reads. Sequencing data are summarized in Online Resource 2: Table S3. The Burrows–Wheeler Aligner (BWA) and NovoAlign software (Novocraft Technologies) were used to align next-generation sequencing reads to the human reference genome GRCh37/hg19. After removal of PCR duplicates, the Short-Read Micro re-Aligner (SRMA) [17] was used to improve variant discovery through local realignments.

### Mutation detection and copy number analysis

To detect somatic mutations, copy number variations, and tumor purity, we used integrated genotyper software (karkinos: http://github.com/genome-rcast/karkinos) as previously reported [3, 20, 51]. For each sample, tumor purity was estimated from allelic imbalance in the matched tumor and normal samples with a program that examined the allelic fractions of heterozygous single nucleotide polymorphisms (SNPs) in regions of loss of heterozygosity (LOH). This algorithm is similar to that described in a previous report from another group [9]. In some cases where LOH regions were not detected, tumor content ratios were estimated from the distribution of mutant allele frequencies. When both calculations failed to estimate tumor cellularity, we presumed it to be 0.2 for the correction of mutant allele frequencies. Somatic mutant allele frequencies adjusted by estimated tumor content ratios, that were ≥15% were retained. Artifacts originating from errors in the sequencing and mapping were also filtered by heuristic filtering and Fisher’s test. To eliminate germline variations in this study, we carried out comparative analyses using paired tumor and normal samples from the same cases for all the samples analyzed, and we extracted the somatic events detected only in tumor tissues. Mutations were validated by Sanger sequencing or RNA sequencing. For validation of mutations, variant allele reads of each RNA-sequencing BAM files were counted using SAMtools v1.2 mpileup (http://www.htslib.org/). Sanger sequencing was also performed for the validation.

To analyze copy number changes, the read depth was compared between normal and tumor for each capture target region. After normalizing by the number of total reads and the GC content bias, the tumor/normal depth ratio was calculated, and values were smoothed using a moving average. Copy number peaks were then estimated using wavelet analysis, and each peak was approximated using complex Gaussian models. A hidden Markov model with calculated Gaussian models was constructed, and copy number peaks were linked to genomic regions. The allelic imbalance for each copy number peak was then calculated, and imbalance information and peak distance were further analyzed by model fitting, yielding integer copy number annotation and tumor purity.

### RNA sequencing

RNA sequencing was performed as previously described [22] for 14 DCG and eight cerebral GBM samples that had RNA of sufficient quality and quantity (Online Resource 2: Table S1). An RNA-sequencing library was prepared using the TruSeq Stranded mRNA LT Sample Prep Kit (Illumina) according to the manufacturer’s protocol. Briefly, 1 µg of total RNA was purified using oligo dT magnetic beads, and poly A+RNA was fragmented at 94 °C for 2 min. cDNA was synthesized using SuperScript II (Invitrogen), and adapter-ligated cDNA was amplified with 12 cycles of PCR. Each library was sequenced using HiSeq 2000, loading four libraries per lane of the flowcell, which produced an average of 59.2 million reads of 101-cycle reads for each sample. RNA-sequencing reads were aligned to a human transcriptome database (UCSC genes) and the reference genome (GRCh37/hg19) using the BWA. If multiple isoforms existed in each annotated gene, the longest isoform was selected. After the transcript coordinate was converted to the genomic position, an optimal mapping result was chosen either from transcript or genome mapping by comparing the minimal edit distance to the reference. Local realignment was then performed within an in-house short reads aligner with small seed size (*k* = 11). Finally, fragments per kilobase of exon per million fragment mapped (FPKM) values were calculated for each UCSC gene while considering strand-specific information. The gene set used in the Gene Set Enrichment Analysis (GSEA) was composed of 320 genes that were up-regulated in “PDGFRA-amplified GBMs” and used in previous reports [34, 38]. The gene set of “Proneural GBMs” was obtained from the GSEA website (http://www.broadinstitute.org/gsea/index.jsp).

### Fusion transcript detection and validation

Fusion analysis was performed with RNA-sequencing data of DCGs in this study and data of 173 GBM samples obtained from The Cancer Genome Atlas (TCGA) website (https://tcga-data.nci.nih.gov). Fastq files from RNA sequencing were used to detect fusion genes using Genomon-fusion (https://genomon-project.github.io/GenomonPagesR/) with default parameters. At least 12 bases matching both sides of the fusion in each read and more than four reads spanning the candidate breakpoint were required to call the fusion transcript. When two sides resided on the same chromosome, we chose a minimum distance of 100,000 bp to reduce read-through transcripts. To validate fusion transcripts, tumor RNA was reverse-transcribed using Superscript III (Invitrogen) according to the manufacturer’s instructions, and the obtained cDNAs were used as PCR templates. Oligo primers for PCR amplification of the three fusion isoforms were designed to amplify only the fusion transcript. Designed primers, the annealing temperature for each set, and the estimated size of the PCR products are shown in Online Resource 2: Table S2. PCR was performed with KOD-Plus-Neo, and optimized thermal conditions were used. PCR products were evaluated on an agarose gel, and purified products were sequenced to validate the presence of the fusion product.

### Microarray data processing

The gene expression microarray data (Affymetrix U133 plus 2.0 platform) reported by Sturm et al. were obtained from National Center for Biotechnology Information (NCBI)’s Gene Expression Omnibus (GEO, http://www.ncbi.nlm.nih.gov/geo) and are accessible through GEO Series accession number GSE36245. These data were normalized to examine the correlation between *SOX10* promoter methylation and expression [48]. Overwrapping expression data with methylation data (GSE36278) was used for correlation analysis.

### Methylation analysis

The Infinium MethylationEpic BeadChip (Illumina) was used to analyze the genome-wide methylation profile of 17 DCGs (Online Resource 2: Table S1) and one non-neoplastic frontal lobe sample as a control following the manufacturer’s instructions. The beta-value was calculated for each CpG site using the following equation as previously reported [3]. Intensity of the methylated allele (M)/[intensity of the unmethylated allele (U) + intensity of the methylated allele (M) + 100] [5]. This beta-value ranged from 0 (unmethylated) to 1 (fully methylated) and reflected the methylation level of each CpG site represented by the probe.

For clustering analysis of methylation data, the Methylation450K BeadChip methylation data from 210 high-grade gliomas and normal cerebellum samples (two normal adult brains and four normal fetal brains) reported by Sturm et al. were obtained from GSE36278 and TCGA website (https://tcga-data.nci.nih.gov) [48]. The information of the tumor region of TCGA samples was obtained from pathological reports in cBioPortal (http://www.cbioportal.org). Methylation data of 224 gliomas including the 14 DCGs in this study were used for clustering analysis. Because three tumor samples (DCG_01, 13, and 14) were determined to have a low tumor content by exome WES data, they were excluded from the clustering analysis. After excluding probes targeting the X and Y chromosomes, and probes associated with an SNP according to TCGA, extraction of common probes between EPIC and 450K probes was performed, and the remaining 300,870 probes in total were used for the following analysis. The standard deviation of beta-values for each probe was calculated, and the top 8000 most variable probes were selected. Unsupervised consensus clustering was then performed utilizing the R package (ConsensusClusterPlus), and the *k*-means algorithm (10 random starting sets, maximum of 1000 iterations) was used to calculate the consensus matrix; *k* = 6 was selected as previously reported [48].

Probes within 1500 bp from the transcription start site (TSS) of protein-coding transcripts (UCSC genes and GRCh37/hg19) were considered to be located in a promoter region, and the mean beta-value of all probes in each promoter for each sample was calculated to represent the promoter methylation status of each gene. To identify genes showing a significantly different methylation status in the promoter between 18 DCGs and 123 cerebral gliomas, the mean beta-value of each promoter for both groups was calculated. Welch’s *t* test and the Benjamini–Hochberg method were used to calculate *p* values and *q* values, respectively. A promoter of a gene was considered to be significantly methylated when the following criteria were fulfilled: *q* values <0.01 and difference >0.2. To validate promoter methylation of significantly methylated genes with additional data, the Methylation450K BeadChip methylation data in Fontebasso et al. (GSE55712), Zhang et al. (GSE50774), and Aihara et al. (JGAS00000000106) [2, 14, 55] were used.

### Motif analysis

A total of 224 samples from the studies of Sturm et al. and from TCGA in addition to the samples of the current study were divided into three groups according to *SOX10* promoter methylation levels (i.e., “*SOX10* promoter hypomethylation” group, beta-value <0.5; “*SOX10* promoter intermediate methylation” group, 0.5 ≤ beta-value <0.7; “*SOX10* promoter hypermethylation” group, beta-value ≥0.7) [48]. To select significantly hypomethylated probes in distal elements (distance from TSS >1500 bp) of the “*SOX10* promoter hypomethylation” group compared to the “*SOX10* promoter hypermethylation” group, the average beta-values of each probe for each group were calculated. *P* values were calculated using Welch’s *t* test, and the Benjamini–Hochberg method was used to calculate *q* values. We chose relatively strict criteria of *q* values <1 × 10^10^ and difference <−0.25 to select nearly top 1000 probes, and a final total of 1070 probes was selected. Windows of 1000 bp around these probes were searched for motifs. De novo motif discovery was performed by using HOMER (v4.9 2-20-2017).

### Statistical analysis

Statistical comparisons of mutated genes were performed using Fisher’s exact test. Overall survival curves were calculated according to the Kaplan–Meier method, and univariate assessment of Kaplan–Meier plots were performed using the log-rank test. Statistical comparisons of gene expression were performed using the Wilcoxon rank-sum test. *P* values less than 0.05 were considered statistically significant.

## Results

### Characteristics of DCGs

We analyzed 27 diffuse gliomas that originated from the cerebellum radiographically (Online Resource 1: Fig. S1). All patients were adults (median age 64 years, range 28–81 years), and WHO histological grades were grade IV in 19 cases, grade III in five, and grade II in three. Detailed patient characteristics are shown in Online Resource 2: Table S1.

The prevalence of common driver gene mutations observed in cerebral gliomas was examined in these 27 DCGs by direct Sanger sequencing (Online Resource 2: Table S4). We found no *IDH*1/2 mutation and only one *TERT* promoter mutation (3.7%) in these DCGs, indicating their distinctive molecular background compared to cerebral gliomas. Although DCGs are located near the brainstem, *H3F3A* K27M mutations were detected in only three cases (11%). We further performed H3 K27M staining for all samples to examine whether K27M was present in other H3 variants. Although *H3F3A* K27M was detected in three cases with Sanger sequencing and these three cases were positive with immunohistochemical staining, all other cases showed negative staining (Online Resource 1: Fig. S2).

### Mutation analysis by WES

**Fig. 1:**
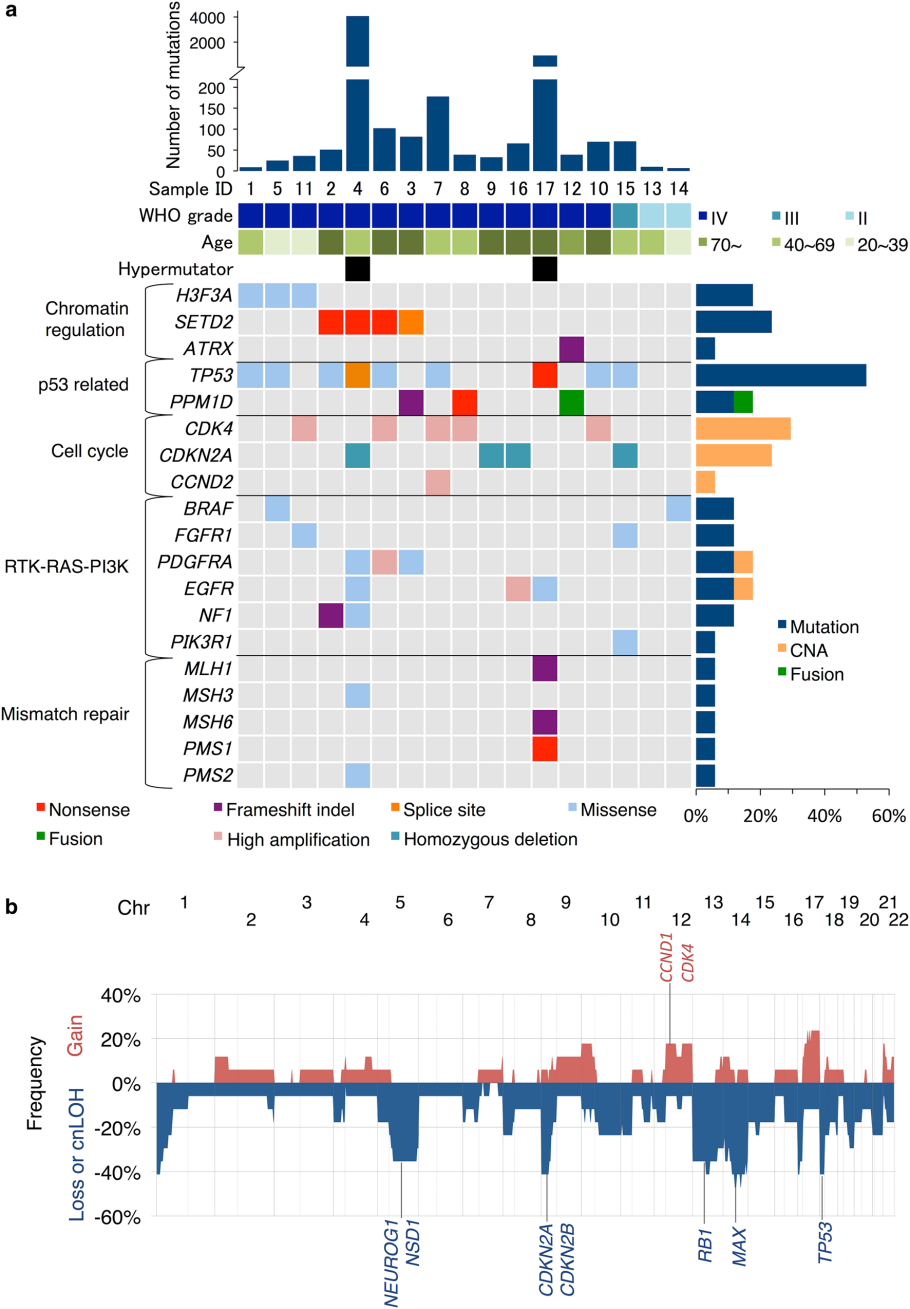
Summary of genomic and chromosomal alterations in DCGs. **a** Representative cancer-related genetic alterations are shown. The number of non-synonymous mutations in each sample, sample ID, WHO grade, age group, and hypermutator are indicated at the *top*. Genes mutated in cancer-related pathways are shown on the *left*. The types of alteration are indicated as *colored boxes*. The frequency of alteration of each gene is shown on the *right*. **b** The overall pattern of CNAs is shown. The *horizontal axis* represents the position on the chromosome. The *vertical axis* represents frequency of gains or losses. Copy-neutral LOH (cnLOH) were counted as loss in this frequency. *Chr* chromosome

To explore driver mutations of DCGs, WES was performed using Illumina HiSeq. Seventeen tumors (14 WHO grade IV, one grade III, and two grade II) and matched normal blood samples were analyzed (Online Resource 2: Table S1). Mean coverage of the coding sequence for tumors and normal blood samples was 122.3× and 97.7×, respectively, with 96.8 and 96.6%, respectively, of bases covered more than 20× (Online Resource 2: Table S3). The tumor content ratio as calculated by the karkinos computational pipeline had a mean value of 61.0%, ranging from 12.0 to 91.3% (Online Resource 2: Table S5). In total, 17,682 tumor-specific somatic mutations were identified, of which 5735 (32.4%) were non-synonymous (Online Resource 2: Table S6). For validation of mutation detection, each position in matched RNA-sequencing reads was examined. Of all positions where non-synonymous mutation was detected, 3021 (2952 substitutions and 69 indels) positions had more than 10 reads in RNA sequencing, and 2647 (2605 substitutions and 42 indels) positions (87.6%) had the same mutation reads, indicating a reasonably high reliability of the WES considering the limited sensitivity of RNA sequencing in detecting mutations. Two cases (DCG_04 and 17) had higher numbers of mutations with loss-of-function mutations in mismatch repair genes, indicating that they were hypermutators (Fig. 1a). The total number of non-synonymous mutations in other non-hypermutator cases was 818 (average 54.5, range 7–178), which included less than 20% of truncating mutations (Online Resource 1: Fig. S3).

The mutational landscape of the 17 DCGs is shown in Fig. 1a and Online Resource 1: Fig. S4. All 35 mutations including five indels in Fig. 1a were validated either by RNA-sequencing reads (28 mutations) or Sanger sequencing (seven mutations) (Online Resource 1: Fig. S5). Among genes related to chromatin regulation, the *H3F3A* K27M mutation, which constitutes a diagnostic criterion for H3 K27M-mutant diffuse midline glioma in the 2016 WHO classification, was observed in three DCGs. All three had pathological features of GBM and were not particularly located near the brainstem compared to other DCGs (Online Resource 1: Fig. S1). Notably, loss-of-function mutations in *SETD2* were found in four GBM cases, and *SETD2* mutations were mutually exclusive with the *H3F3A* K27M mutation. *SETD2* regulates chromatin function by methylating histone H3K36. However, no mutation in other genes responsible for H3K36 mono- or di-methylation such as *NSD1*-*3*, *ASH1L*, or *SMYD2* was detected except for in the two hypermutator cases.

Disruption of p53 function was common in DCGs, and *TP53* was the most frequently mutated gene (58.9%). Activation of *PPM1D* suppresses p53 function [41, 55]. The activating truncation mutation of *PPM1D* was found in two cases, and was mutually exclusive with *TP53* mutation.

*FGFR1* mutation in the tyrosine kinase domain, which has been reported in pilocytic astrocytomas, diffuse brainstem gliomas, and thalamic gliomas, was identified in two DCGs (DCG_11 and _15) [14, 19]. Both cases had p.K656E mutation, and p.V472M mutation was also found in DCG_11. *FGFR1* mutation harboring p.K656E, whose biological effect in promoting tumor growth has been demonstrated, has been reported in pediatric diffuse midline glioma H3 K27M-mutant and pilocytic astrocytomas [14, 19, 24]. *BRAF* mutations, which included the p.V600E mutation in DCG_14, were detected in two cases. *PDGFRA* extracellular domain mutations, which potentially disrupt ligand interaction, were identified in two cases [38]. Although *EGFR* mutations were found in two hypermutators (p.E451K in DCG_04 and p.T680M in DCG_17), these mutations were not located at positions where mutation has been frequently observed in GBMs [6].

### Copy number aberrations (CNAs)

Copy number variations estimated from WES data revealed frequent loss of chromosomes 1p, 5q, 9p, 13, 14, 17p, and 18p (Fig. 1b; Online Resource 1: Fig. S6). Homozygous deletion of *CDKN2A* located on chromosome 9p was observed in four cases. Also, focal high amplification of *CDK4* (*n* = 5), *CCND2* (*n* = 1), *EGFR* (*n* = 1), and *PDGFRA* (*n* = 1) was observed (Fig. 1a). Amplification of *EGFR*, gain of chromosome 7, and loss of chromosome 10 were infrequent in adult DCGs compared to large-scale genetic analysis data of GBMs [6].

### *SETD2* mutation and H3K36 trimethylation

**Fig. 2:**
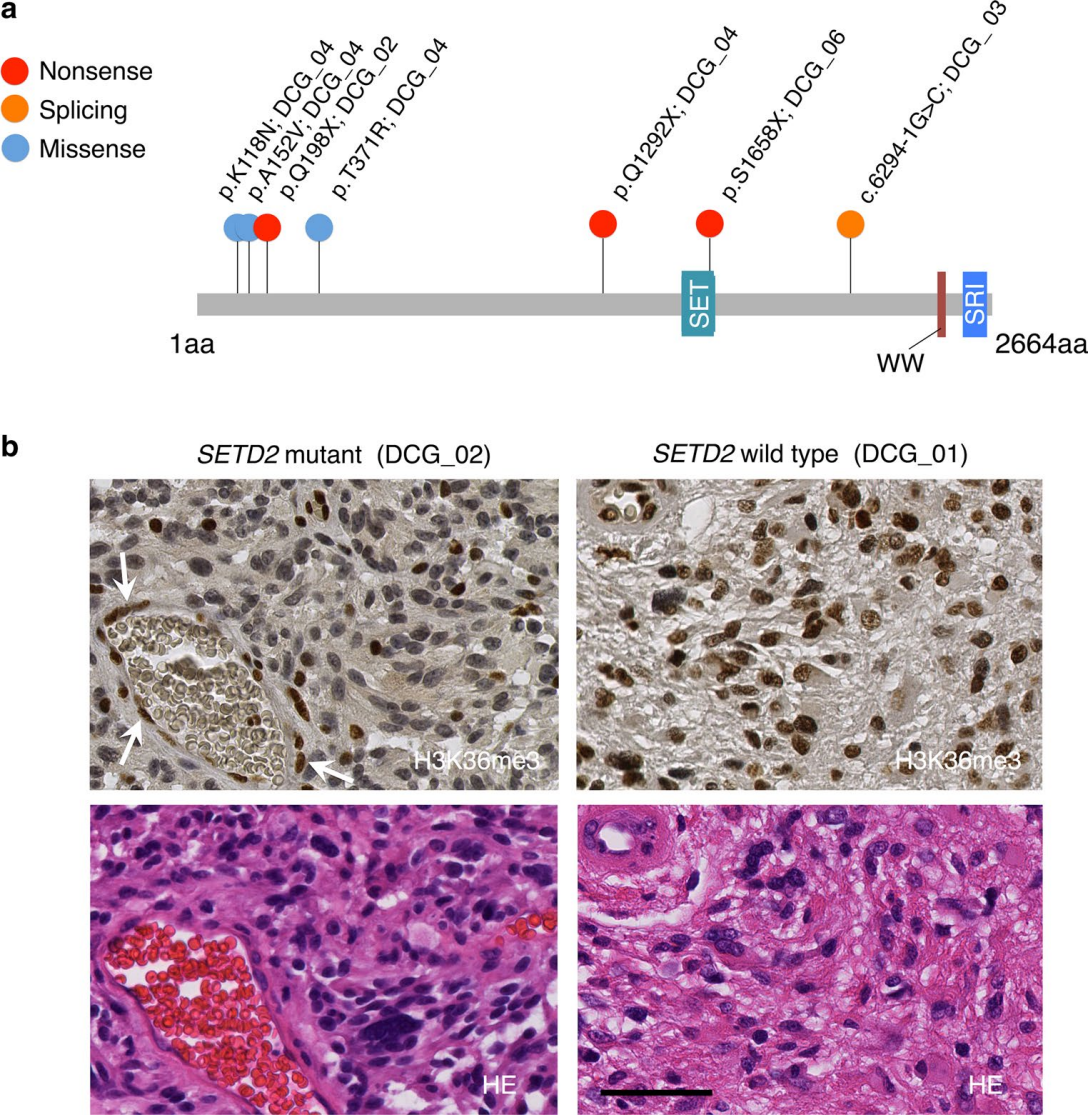
Somatic mutations in *SETD2.* **a** Distribution of *SETD2* alterations identified in four cases. The types of mutations are *color-coded* as indicated. Amino acid changes and the case IDs are shown on *top*. Protein domains are depicted as *colored boxes* with an abbreviated domain name. *SET* SET domain, *WW* WW domain, *SRI* SRI domain, *aa* amino acid. **b** Representative immunohistochemical staining of H3K36 trimethylation (H3K36me3) and hematoxylin and eosin (HE) staining of *SETD2* mutant (DCG_02) and wild-type (DCG_01) DCGs. Nuclei of vascular endothelial cells are indicated by the *white arrows* as the internal positive control for H3K36me3 staining. *Scale bar* 50 μm

Truncating mutations of *SETD2* were identified in four of 17 DCGs; three were nonsense mutations (p.Q1292X, p.S1658X, and p.Q198X), and one was a splicing site mutation that caused a frameshift (Figs. 1a, 2a). A hypermutator (DCG_04) with a nonsense *SETD2* mutation (p.Q1292X) also had three missense *SETD2* mutations (p.K118N, p.A152V, and p.T371R). All of these *SETD2* mutations were found in GBM; four of 14 grade IV gliomas had *SETD2* mutation. Patients with *SETD2* mutation were relatively older than those with *H3F3A* K27M mutation (69 ± 17 vs. 42 ± 19 years of age). *SETD2* is the only known gene that can catalyze H3K36 trimethylation, and loss-of-function mutation of *SETD2* eventually decreases H3K36 trimethylation and contributes to tumor development [11, 23, 44, 56]. Immunohistochemical analysis to evaluate whether H3K36 trimethylation was impaired in *SETD2* mutant DCGs indicated that H3K36 trimethylation was indeed reduced in tumors with *SETD2* mutation compared to tumors with wild-type *SETD2*, in which no decrease in H3K36 trimethylation was observed (Fig. 2b; Online Resource 1: Fig. S7).

### *PPM1D* mutation and a novel *PPM1D* fusion

**Fig. 3:**
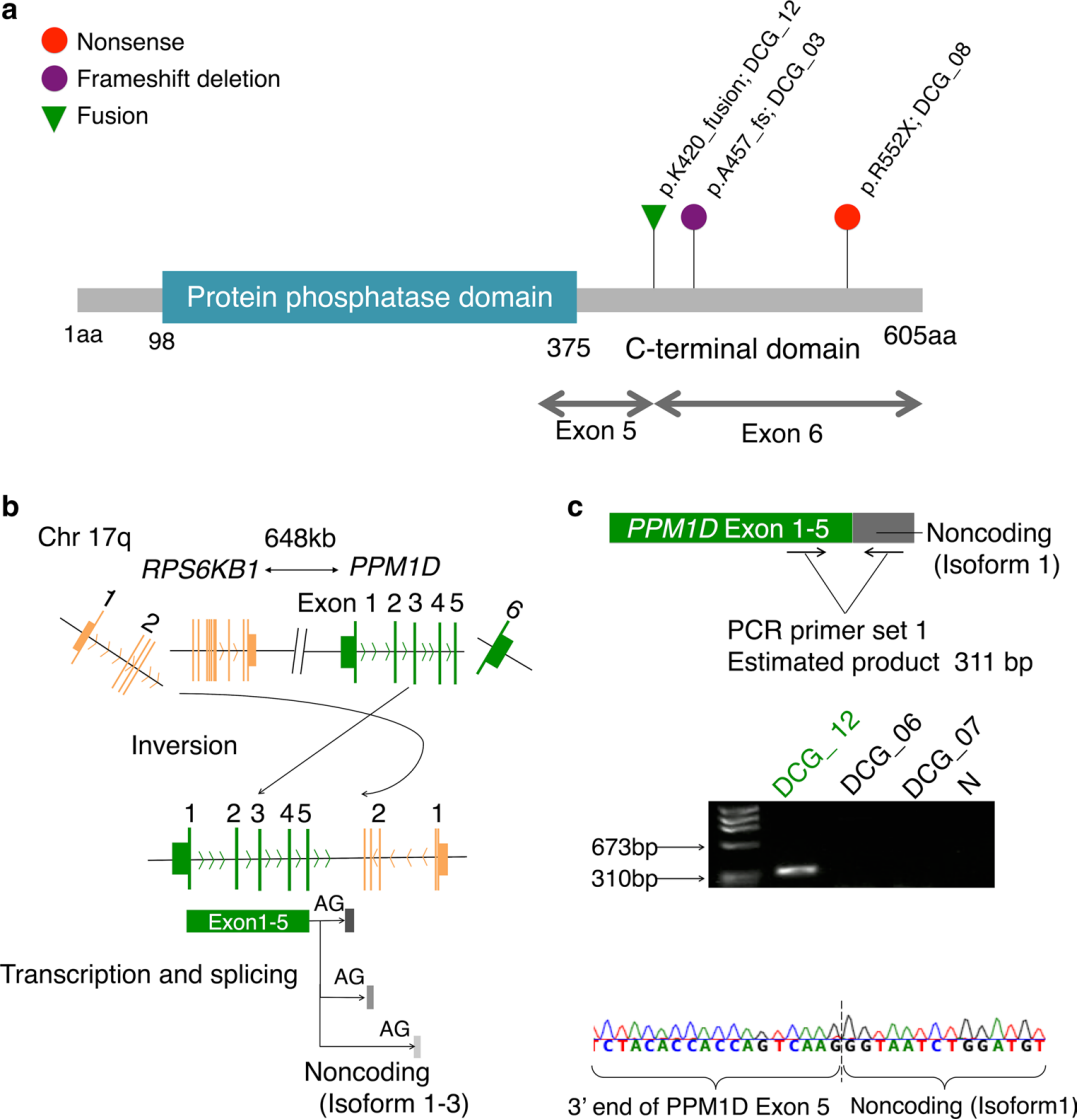
Gain-of-function mutations and fusion *PPM1D.* **a** Distribution of *PPM1D* alterations identified in DCGs. The types of mutations and a fusion are *color-coded* as indicated. The protein phosphatase domain is depicted as a *colored box*. All alterations were in the C-terminal domain without affecting the protein phosphatase domain. Amino acid changes and case IDs are shown on top. *Chr* chromosome, *aa* amino acid. **b** Scheme of a chromosomal inversion in DCG_12. The new oncogenic transcript lost the C-terminal domain in *PPM1D* exon 6, leaving the protein phosphatase domain in exons 1–5, which underwent fusion with an intragenic region of *RPSK6B1* as a consequence of a chromosomal inversion, giving rise to three fusion isoforms as shown. **c** Validation PCR for isoform 1 of the *PPM1D*-noncoding (antisense RPS6KB1 isoform 1) fusion. The PCR primers were designed to specifically amplify fusion products (*top*). A PCR band of the estimated size was detected in DCG_12 (*middle*). In the negative control lane (N), PCR product was amplified without template DNA and electrophoresed. The predicted sequence was confirmed by Sanger sequencing (*bottom*)

Truncating mutations in the final exon of *PPM1D* (p.457_465del and p.R552X) were found in two DCGs; these mutations were previously reported as gain-of-function mutations in brainstem glioma harboring *H3F3A* K27M [55] (Figs. 1a, 3a). However, unlike those brainstem gliomas, neither of our cases had simultaneous *H3F3A* K27M mutation. In addition, a novel *PPM1D* fusion was discovered in another DCG (DCG_12) by fusion analysis of RNA-sequencing data (Fig. 3a, b; Online Resource 2: Table S7). RNA-sequencing reads in DCG_12 strongly suggested the presence of fusion transcripts between the 3′-end of exon 5 of *PPM1D* and reciprocal noncoding products derived from an intragenic region of *RPSK6B1*, which were produced as a consequence of a chromosomal inversion (Fig. 3b; Online Resource 1: Fig. S8a). These noncoding products consisted of a few isoforms that were all followed by consensus sequences of a splice acceptor site (AG). All of these fusion transcripts had lost the C-terminal regulatory domain of *PPM1D*, although they retained the protein phosphatase catalytic domain intact, indicating that this fusion may have acquired gain-of-function properties by a mechanism similar to the one reported for the truncating mutation of this gene (Fig. 3b) [41, 55]. Three isoforms of these predicted fusion products were validated by direct sequencing following PCR amplification of the cDNA (Fig. 3c; Online Resource 1: Fig. S8b). This type of *PPM1D* fusion was not detected in RNA-sequencing data of 173 TCGA GBMs, which mostly consisted of cerebral GBMs (Online Resource 2: Table S8).

In total, we identified alterations suppressing p53 function in 71% (12/17) of DCGs, *PPM1D* alterations in three DCGs, and *TP53* mutations in nine DCGs.

### Global DNA methylation profiling

**Fig. 4:**
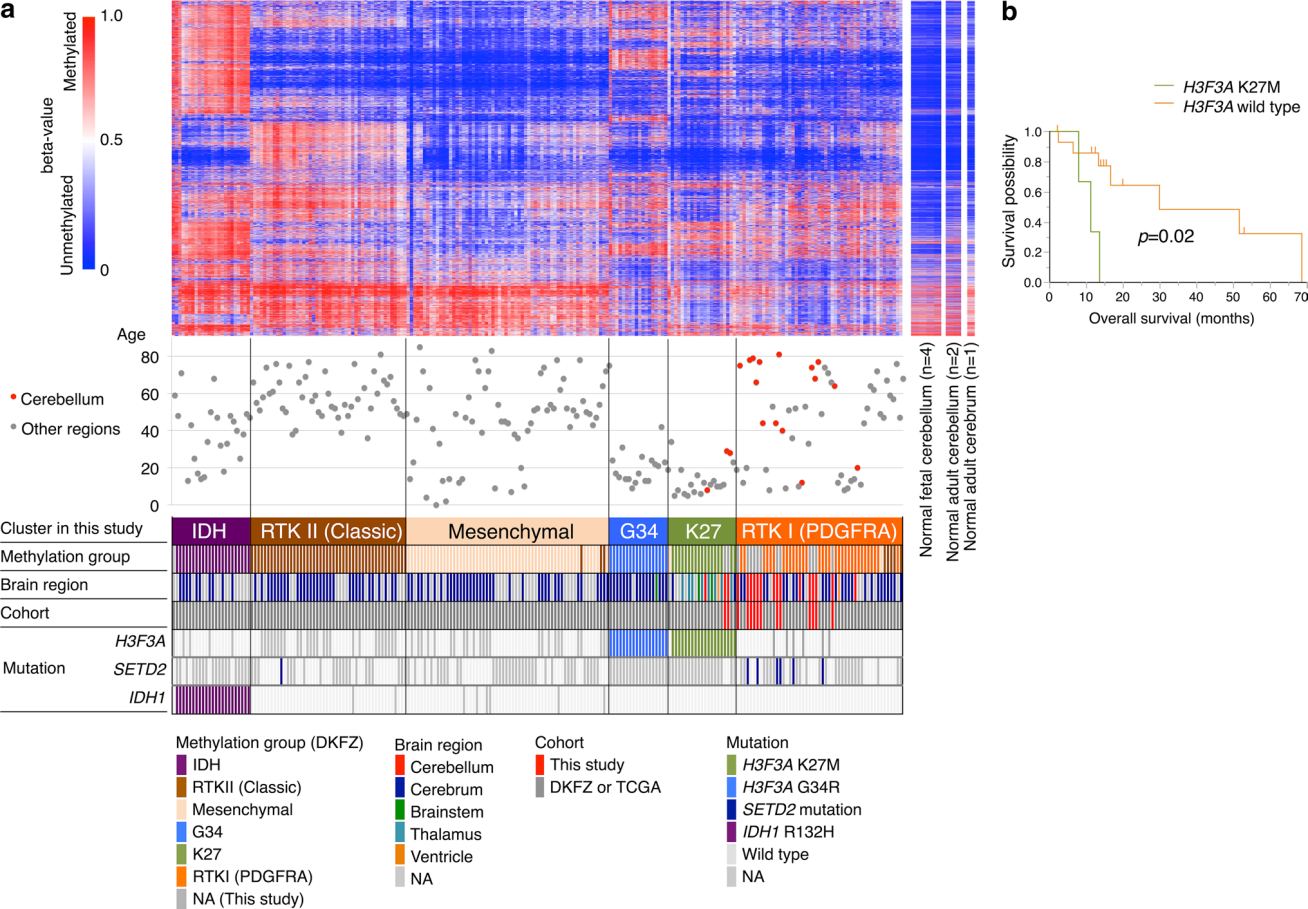
Methylation profiling of DCGs. **a** Heatmap of the methylation level in 14 DCGs in this study, 210 high-grade gliomas of previous studies, and control samples. Unsupervised *k*-means clustering with 224 tumor samples was performed using the top 8000 variant probes. Patients’ age, methylation cluster in this study and in a previous report [48], brain region, cohorts of each sample, and the mutational states of three genes are shown *below*. All of our samples and four cerebellar gliomas of previous reports were in either the “K27” group or the “RTK I” group. **b** Kaplan–Meier analysis of overall survival for three DCGs with *H3F3A* K27M and 16 *H3F3A* wild-type cerebellar GBMs

To clarify epigenetic characteristics of DCGs, the genome-wide DNA methylation profile of 17 DCGs (Online resource 2: Table S1) was analyzed using the InfiniumEpic array platform. To compare the methylation profile of DCGs with that of gliomas that originated from other regions, clustering analysis was performed together with Infinium data of 210 high-grade gliomas in the German Cancer Research Center (DKFZ) methylation study [48]. Three DCGs (DCG_01, 13, and 14) were excluded from the clustering analysis because they showed a very similar methylation profile to normal brain, probably due to their low tumor content (less than 15%) (Online Resource 2: Table S5). Consistent with the previous study, six methylation clusters were identified in our analysis (Fig. 4a; Online Resource 1: Fig. S9) [48]. Notably, all DCGs were clustered into either of two methylation groups; two DCGs with *H3F3A* K27M mutation were clustered together with diffuse midline glioma, K27M-mutant (the “K27” group), whereas all of the other 12 DCGs were clustered in the “RTK I (PDGFRA)” group. In addition, all four cerebellar gliomas identified in the 210 high-grade gliomas analyzed together with our data were clustered into the “K27” or the “RTK I” group. These results indicated that only two methylation groups are present in DCGs depending on the presence or absence of *H3F3A* K27M mutation. Survival analysis to evaluate the prognostic significance of these methylation groups showed that cerebellar GBM with *H3F3A* K27M mutation had significantly shorter overall survival (*p* = 0.02) than *H3F3A* wild-type cerebellar GBM (Fig. 4b), even though the former group included younger patients who generally have longer survival time with GBM than the latter group (median age; 42 ± 19 vs. 62 ± 17 years of age). This result suggested that *H3F3A* K27M mutation may be a poor prognostic factor of DCGs as previously demonstrated in pediatric diffuse intrinsic pontine glioma [21].

### Gene expression profiling

**Fig. 5:**
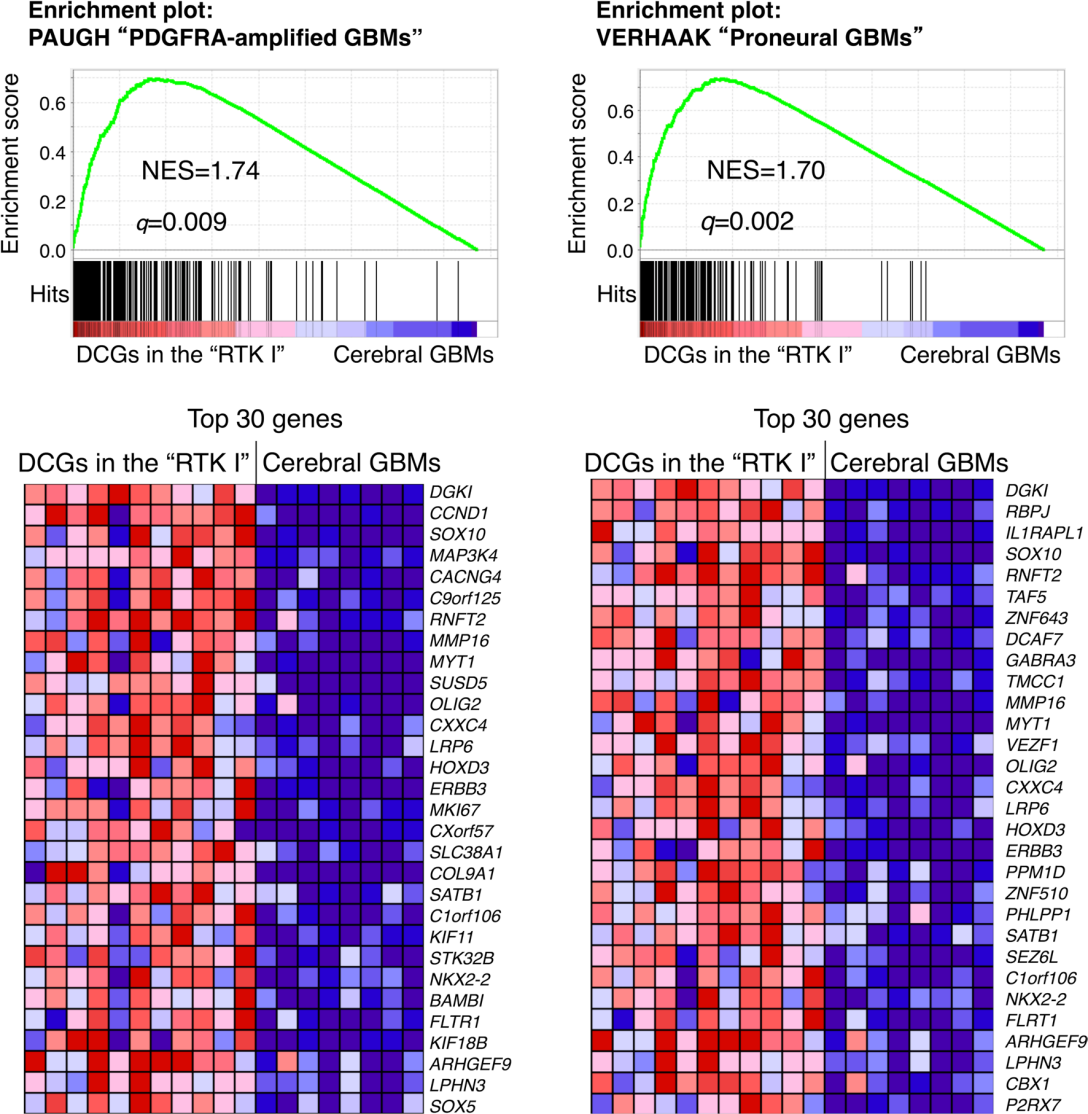
Gene expression analysis of DCGs and cerebral GBMs. GSEA showed that two gene sets were up-regulated in 11 DCGs in the “RTK I” group compared with eight cerebral GBMs. One gene set was overexpressed in “PDGFRA-amplified GBMs” (*left*), and the other gene set was overexpressed in “Proneural GBMs” (*right*). The false discovery rate (*q*) and the normalized enrichment score (NES) are shown (*top*). The top 30 significantly up-regulated genes of each gene set in DCGs are shown (*bottom*)

Because all DCGs without H3 K27M mutation in this study were clustered in the “RTK I” methylation group, we next examined whether DCGs in this “RTK I” group have a similar gene expression pattern to other gliomas in this group, which often have *PDGFRA* amplification [48]. Gene Set Enrichment Analysis demonstrated that compared with adult cerebral GBMs (*n* = 8) that we analyzed by RNA sequencing, DCGs in the “RTK I” group (*n* = 11) were enriched for the gene set associated with the signature of “PDGFRA-amplified GBMs” (*q* = 0.009, normalized enrichment score (NES) = 1.74) and of “Proneural GBMs” (*q* = 0.002, NES = 1.70) described in the TCGA study (Fig. 5) [34, 52]. A similar trend in gene set enrichment was also obtained even when DCGs in both the “RTK I” group and the “K27” group (*n* = 14) were compared with cerebral GBMs (*n* = 8) (Online Resource 1: Fig. S10). Significantly up-regulated genes in these DCGs included transcriptional factors important for oligodendroglial development such as *SOX10*, *OLIG2*, *NKX2*-*2*, *SOX5*, and *ERBB3* (Fig. 5; Online Resource 1: Fig. S10) [37, 40].

### Differentially methylated and expressed genes in DCG

**Fig. 6:**
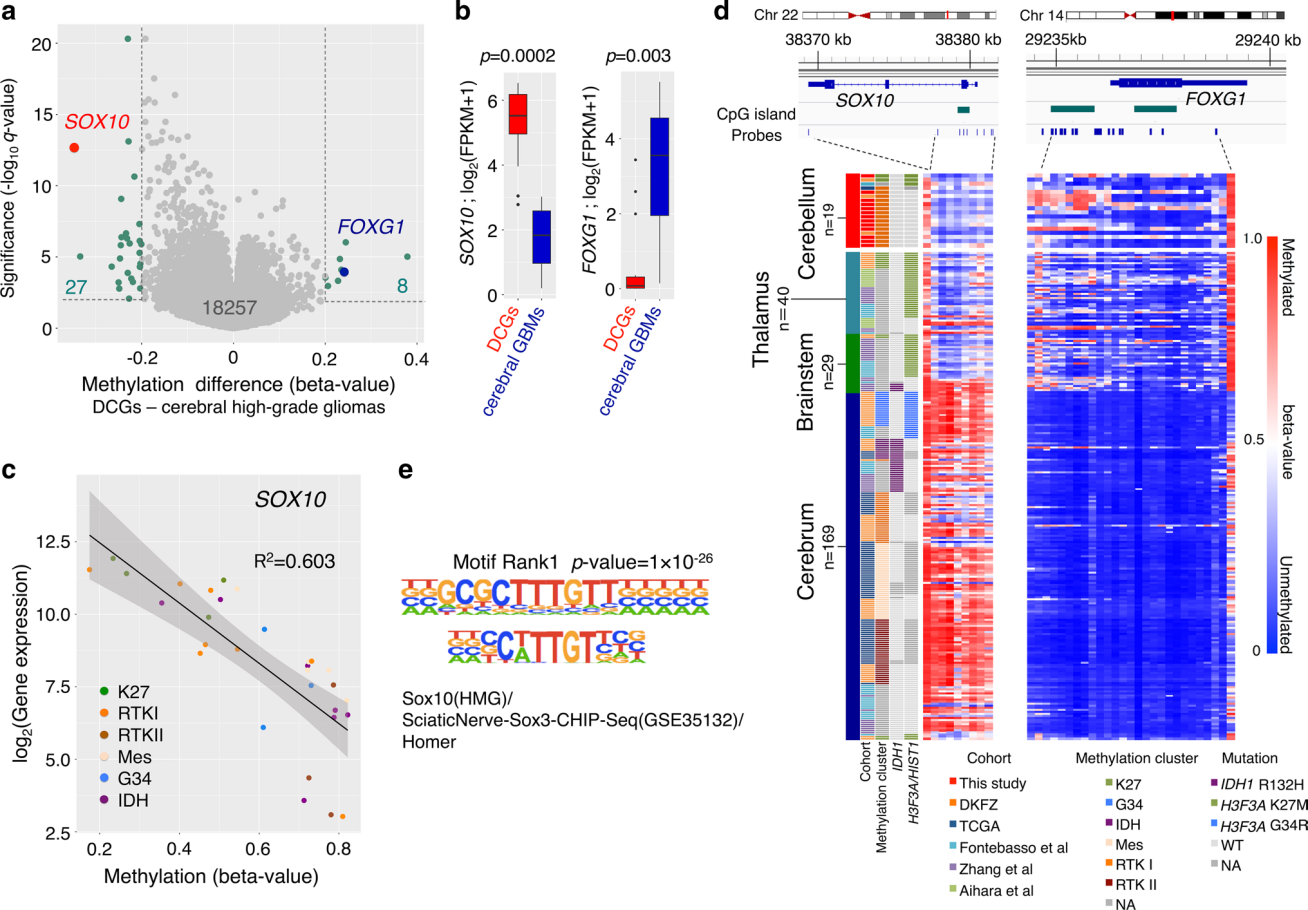
Differentially methylated and expressed genes in DCGs. **a** A volcano plot comparing DNA promoter methylation between 18 DCGs and 123 cerebral high-grade gliomas is shown. *One dot* represents one gene. The *q* values that were calculated using a paired two-sided moderated Welch’s *t* test were plotted on the *y* axis. Methylation differences expressed as beta-values are plotted on the *x* axis. The methylation level of a gene was considered to be significantly different when the *q* value was <0.01 and the methylation difference was >0.2. **b** *Boxplot* of *SOX10* and *FOXG1* expression values obtained from RNA sequencing; 14 DCGs and eight cerebral GBMs were compared. Differences in gene expression for the target genes were analyzed using the Wilcoxon rank-sum test. **c** Inverse correlation of promoter methylation and gene expression of *SOX10* is shown using data of a previous study [48]. *R* denotes Pearson’s correlation coefficient. **d** Methylation level of *SOX10* and *FOXG1* promoters in 257 samples. These samples were cases in this study and cases in five previously published studies for which we obtained information of tumor regions. A map of the chromosomes (Chr) of these two genes and the positions of the Infinium probes are shown at the *top*. *Each row* represents a sample, and each *vertical bar* represents an Infinium probe. Anatomical brain regions of tumors are shown on the *left*. Cohort, methylation cluster in this study, and mutational status of the two genes are also shown. **e** The most enriched motif in sequences around 1070 hypomethylated probes of the “*SOX10* promoter hypomethylation” group is shown at the top (*p* value = 1 × 10^−26^). The consensus SOX10 motif is shown at the *bottom*

Next, to identify region-related gene promoter methylation of DCGs, Infinium data were compared between 18 DCGs and 123 cerebral gliomas. A volcano plot showed a general shift towards hypomethylation in DCGs (Fig. 6a), and *SOX10* was one of the most significantly hypomethylated genes in DCGs (Fig. 6a and Online Resource 2: Table S9). *SOX10* is a key transcription factor in oligodendrocyte precursor cells and regulates *PDGFRA* [13, 40]. Significantly higher gene expression of *SOX10* in DCGs, compared with that in cerebral gliomas (*n* = 8) (*p* = 0.0002), was concordant with the promoter hypomethylation of this gene (Fig. 6b). An inverse correlation of promoter methylation and *SOX10* expression was also observed in previously reported data (*R* ^2^ = 0.603) (Fig. 6c). Notably, hypomethylation of the *SOX10* promoter was evident not only in all DCGs regardless of *H3F3A* mutation status, but also in brainstem and thalamic gliomas with *H3F3A* K27M (Online Resource 1: Fig. S11). On the other hand, *FOXG1*, an important neuronal lineage marker, was one of the most hypermethylated genes in our comparison (Fig. 6a; Online Resource 2: Table S9). In accordance with the promoter hypermethylation, the expression level of *FOXG1* in DCGs was significantly lower than that in cerebral gliomas (Fig. 6b). Sturm et al. previously demonstrated significantly higher promoter methylation and lower expression of *FOXG1* in brainstem and thalamic gliomas with *H3F3A* K27M [48]. Although cerebral gliomas in the “RTK I (PDGFRA)” group also showed comparatively lower methylation of the *SOX10* promoter, these cerebral tumors did not show hypermethylation in the *FOXG1* promoter, unlike cerebellar gliomas and brainstem and thalamic gliomas with *H3F3A* K27M (Online Resource 1: Fig. S11). *OLIG1* and *OLIG2*, which are other oligodendroglial markers that are highly methylated in gliomas with *H3F3A* G34R/V, were not methylated in DCGs (Online Resource 1: Fig. S11) [48].

To validate methylation changes in the promoters of *SOX10* and *FOXG1* in brainstem and thalamic gliomas, three other data sets, methylation data of 32 thalamic gliomas, 26 brainstem gliomas, and 46 cerebral gliomas obtained from previous reports, were combined and analyzed with our data (Fig. 6d) [2, 14, 55]. This analysis also demonstrated *SOX10* promoter hypomethylation and *FOXG1* promoter hypermethylation in K27M-mutant gliomas of the brainstem and thalamus as well as of DCGs compared to gliomas in other regions.

### Enrichment of the SOX motif in hypomethylated DNA regions

We then examined whether SOX10 does indeed influence global gene expression in gliomas such as DCGs that have higher *SOX10* expression. We divided 224 samples into three groups depending on the methylation level of the *SOX10* promoter (Online Resource 1: Fig. S12a). Most of the gliomas categorized in the “*SOX10* promoter hypomethylation” group consisted of DCGs, thalamic and brainstem gliomas in the “K27” group, and some of the cerebral gliomas in the “RTK I” group. The other cerebral gliomas that were grouped in the “IDH”, “Mesenchymal”, “RTK II”, or “G34” groups were categorized in the “*SOX10* promoter intermediate methylation” group or “*SOX10* promoter hypermethylation” group. We performed motif analysis for the non-promoter probes because lineage-specific transcription factors typically regulate gene expression by binding distal regulatory elements known as enhancers [30]. In this analysis, a de novo motif scan demonstrated that the most enriched motif in the sequences around hypomethylated probes of the “*SOX10* promoter hypomethylation” group was the sequence: CNTTGTT, which may possibly be bound by SOX family transcription factors including SOX10 (Fig. 6e; Online Resource 1: Fig. S12a, S12b and S12c) [46].

## Discussion

The genetic analyses in this study supported the concept that the molecular characteristics of adult DCGs are different from those of common cerebral gliomas. Frequent gene alterations observed in adult cerebral GBMs such as mutations in the *TERT* promoter, *PIK3CA*, *PTEN*, and *RB1* were not detected in DCGs, and the rates of chromosome 10 loss, chromosome 7 gain, and mutation or amplification of *EGFR* were much lower than those of common cerebral high-grade gliomas [4, 6]. The *IDH1* mutation, which is very frequent in diffuse lower-grade gliomas [8, 29], was rare in DCGs. In addition, subsequent integrated omics analysis in the present study clearly demonstrated the brain region-related distinct characteristics of DCGs.

WES analysis identified recurrent loss-of-function mutation of *SETD2* in DCGs. All *SETD2* mutations were present in GBMs that had neither the *H3F3A* K27M nor the G34R/V mutation. A previous report demonstrated that the *SETD2* mutation frequently observed in pediatric GBM located in cerebral hemispheres occurred mutually exclusively with *H3F3A* G34R/V mutation [15]. In this study, we showed that *SETD2* mutation was also frequent in DCGs in elderly adults. The frequency of *SETD2* mutation (24%, 4/17) in DCGs was significantly higher than those in previous large-scale genetic analyses of adult gliomas, which showed a *SETD2* mutation rate of 1.7% in GBM (5/292, *p* = 0.0007) and 2.1% in lower-grade glioma (3/283, *p* = 0.0002) [6, 8]. *SETD2* mutation was quite rare in previous reports analyzing brainstem or thalamic gliomas in either adults or children [14, 15, 50, 54, 55].

In addition to pediatric cerebral GBM, inactivating mutation of *SETD2* has been reported as a driver gene mutation in clear cell renal cell carcinoma (ccRCC), leukemia, and breast cancer [11, 23, 56]. The study of ccRCC demonstrated that *SETD2* mutation causes loss of H3K36 trimethylation and consequently leads to altered chromatin accessibility and widespread defects in transcript processing that eventually result in promotion of cancer development [23, 44]. Like in other cancers, reduced H3K36 trimethylation in DCGs with *SETD2* mutation was confirmed by immunohistochemistry [39], indicating that epigenetic regulation was altered in these tumors. Because *H3F3A* K27M mutation, which we found in three DCGs, also results in the loss of H3K27 trimethylation, such epigenetic alterations may play major roles in the pathogenesis of DCG. Recent studies have identified potential drugs targeting epigenetic alterations such as *H3F3A* K27M [28, 36]. Another study showed that the WEE1 inhibitor selectively kills H3K36-deficient cancers through dNTP starvation resulting from ribonucleotide reductase subunit M2 depletion [35]. Therefore, assessment of these mutations may lead to new drugs for patients with these ominous diseases in the future.

We showed that the p53 pathway is frequently disrupted, and that *PPM1D* is one of the recurrently altered genes in DCGs. The protein encoded by *PPM1D* is a p53-dependent serine/threonine protein phosphatase that negatively regulates molecules such as p53, CHK2, H2AX, and ATM, which are related to cell stress response pathways. High DNA copy-number amplification or overexpression of *PPM1D* has been detected in several tumors including breast cancer, ovarian cancer, and medulloblastoma [7, 27, 49]. Mosaic *PPM1D* truncating mutation, which is found in the germline DNA of a small population of breast or ovarian cancer patients, was recently determined to be a genetic risk factor for those cancers [41]. Such truncation was shown to enhance *PPM1D* stability, and consequently, works as a gain-of-function oncogenic mutation. A previous report demonstrated that a similar somatic truncating mutation was also frequent in brainstem glioma, and that *PPM1D* truncation and *TP53* mutation were found mutually exclusively in six and 19 samples, respectively, in 33 brainstem gliomas, whereas *PPM1D* truncation was detected in only one of 57 cerebral gliomas and was absent in thalamic glioma [55]. In this study, we identified a novel *PPM1D* fusion, in addition to the truncating mutations in exon 6, neither of which has been previously reported in cerebellar gliomas. This fusion is a novel mechanism of *PPM1D* alteration that was identified by RNA sequencing, but should have been missed by WES only. Therefore, the same mutations may exist in brainstem gliomas and other cancers if appropriately examined. Because *PPM1D* alterations have been a target of drug development, novel therapeutic opportunities may be available in the future for cerebellar and brainstem gliomas with *PPM1D* truncating mutation or fusion [12].

Some transcription factors play critical roles in the determination of cell fate. For example, SOX10, which is repressed by polycomb repressor in neural stem cells and is induced in oligodendroglial precursor cells, is a key transcription factor for the oligodendroglial lineage [37, 40]. In this study, we demonstrated that the CpG island promoter methylation status of such developmental genes, particularly of *SOX10* and *FOXG1*, was remarkably different between gliomas that originated from different regions. Furthermore, DCGs were characterized by hypomethylation of the *SOX10* promoter and hypermethylation of the *FOXG1* promoter regardless of the presence or absence of K27M mutation, which resulted in upregulation of SOX10 (SOX10+) and downregulation of FOXG1 (FOXG1−). Previously, Sturm et al. showed that epigenetic silencing of *FOXG1* was characteristic of diffuse midline gliomas that are H3 K27M-mutant and located in the brainstem or thalamus, and that this type of glioma has a distinct cell of origin characterized by OLIG1+, OLIG2+, and FOXG1− [48]. Notably, our analysis revealed not only that the status of OLIG1+, OLIG2+, and FOXG1− was shared between DCGs and K27M midline gliomas, but also that *SOX10* promoter hypomethylation and consequent gene overexpression was commonly found among these tumors, whereas *SOX10* expression is repressed by promoter hypermethylation in most other cerebral high-grade gliomas. Because most of the tumor-specific targets of de novo CpG island methylation are genes that are silenced by the polycomb repressor, hypermethylation of promoter CpG islands in key developmental transcription factor genes in tumors may reflect their repressed status in the tumor’s tissue of origin; thus, the methylation status of these genes may reflect the regulation of dominant transcription factors during their developmental course [42, 45, 53]. In that regard, we think that it is especially interesting that DCGs and K27M midline gliomas had a similar methylation pattern in the promoters of key developmental transcription factors such as SOX10, FOXG1, OLIG1, and OLIG2, suggesting a particular commonality in their cell of origin or tumor developmental process that appears to be distinct from other cerebral gliomas.

In contrast to the similarity in the methylation status of CpG islands of the developmental transcription factors, our global methylation profile analysis of adult DCGs demonstrated that all DCGs were clustered into either the “K27” group or the “RTK I (PDGFRA)” group, indicating two representative epigenetic profiles are present in adult DCGs. In accordance with these methylation patterns, gene expression analysis demonstrated that adult DCGs were significantly enriched for the PDGFRA-associated genes that were observed in the “PDGFRA-amplified GBMs” in the TCGA project; these GBMs were mostly classified as the “Proneural type” GBMs based on their gene expression profile [34, 52]. Upregulation of *SOX10*, which positively regulates *PDGFRA* in the oligodendroglial lineage, may explain why DCGs showed the “RTK I (PDGFRA)” methylation pattern [13, 40], whereas only a few had *PDGFRA* amplification. Indeed, diffuse intrinsic pontine gliomas, which often harbor H3 K27M mutation and *SOX10* upregulation, also frequently show higher expression of *PDGFRA* and a specific PDGFRA-related gene expression signature, indicating that the PDGFRA-related gene expression signature is shared by “K27” gliomas and DCGs categorized in the “RTK I (PDGFRA)” methylation group [33, 34]. Nonetheless, it is noteworthy that the prognosis of patients with DCG was quite different between the “K27” group and the “RTK I (PDGFRA)” group, thus emphasizing the clinical importance of distinguishing these two groups.

In summary, we demonstrated that compared to most cerebral gliomas, adult DCGs had characteristic genetic alterations and epigenetic profiles, which included frequent *SETD2* and *PPM1D* alteration and *PDGFRA*-related genetic and epigenetic signatures, and that these DCGs were characterized by upregulation of SOX10 and downregulation of FOXG1, which possibly reflects their cell of origin and developmental course. Notably, such a characteristic expression pattern of developmental transcription factors was commonly observed in diffuse midline glioma H3 K27M-mutant, which is a newly defined entity in the 2016 WHO classification of brain tumors. We think that further studies will clarify differences in the cell of origin among tumors that originated from different brain regions and refine the tumor classification, and that tailored therapy that considers tumor molecular characteristics related to the tumor region will be available in the future.

## Electronic supplementary material

Below is the link to the electronic supplementary material.

Supplementary material 1 (PDF 61749 kb)

Supplementary material 2 (XLSX 1119 kb)
