# Supplementary material for: Distinct molecular profile of diffuse cerebellar gliomas
Source: Acta Neuropathol. 2017 Aug 29;134(6):941–56. doi: 10.1007/s00401-017-1771-1 (PMC5663812; doi:10.1007/s00401-017-1771-1)
Supplement: Supplementary file 1 — Supplementary material 1 (PDF 61749 kb) [file 401_2017_1771_MOESM1_ESM.pdf]

Online resource 1 : Supplementary figures

Journal name:

Acta Neuropathologica

Title:

Distinct molecular profile of diffuse cerebellar gliomas

Authors:

Masashi Nomura, Akitake Mukasa, Genta Nagae, Shogo Yamamoto, Kenji Tatsuno, Hiroki Ueda, Shiro Fukuda, Takayoshi Umeda, Tomonari Suzuki, Ryohei Otani, Keiichi Kobayashi, Takashi Maruyama, Shota Tanaka, Shunsaku Takayanagi, Takahide Nejo, Satoshi Takahashi, Koichi Ichimura, Taishi Nakamura, Yoshihiro Muragaki, Yoshitaka Narita, Motoo Nagane, Keisuke Ueki, Ryo Nishikawa, Junji Shibahara, Hiroyuki Aburatani, Nobuhito Saito

Corresponding author:

Akitake Mukasa

Department of Neurosurgery, Graduate School of Medicine, The University of Tokyo

E-mail: mukasa-nsu@umin.ac.jp

Hiroyuki Aburatani

Genome Science Division, Research Center for Advanced Science and Technology (RCAST), The University of Tokyo

E-mail: haburata-tky@umin.ac.jp

Fig. S1 – S12

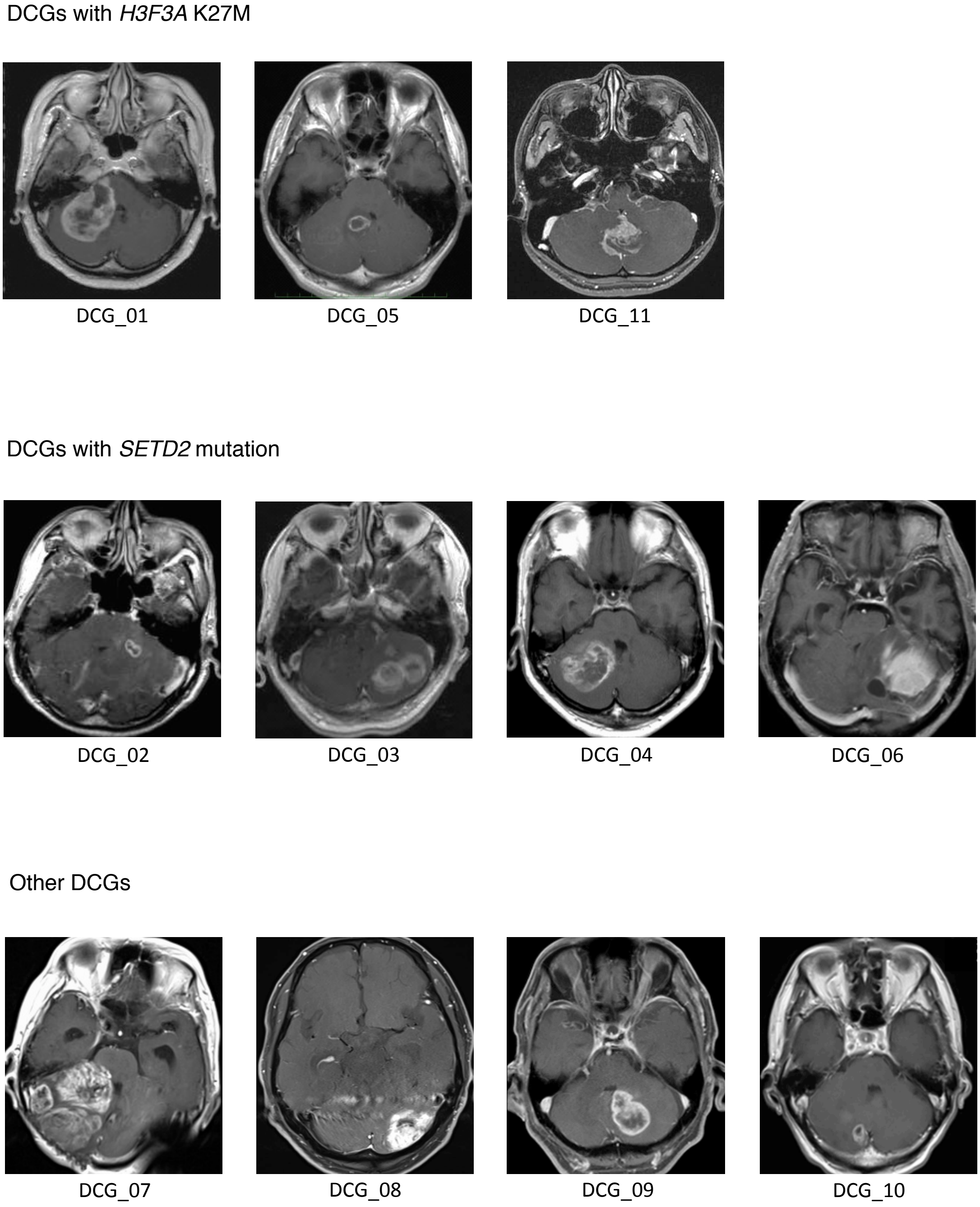

Fig. S1 Magnetic resonance imaging (MRI) findings of representative DCGs**.** Gadolinium-enhanced T1-weighted axial MRI of three DCGs with *H3F3A* K27M, four DCGs with *SETD2* mutation, and four other DCGs are shown.

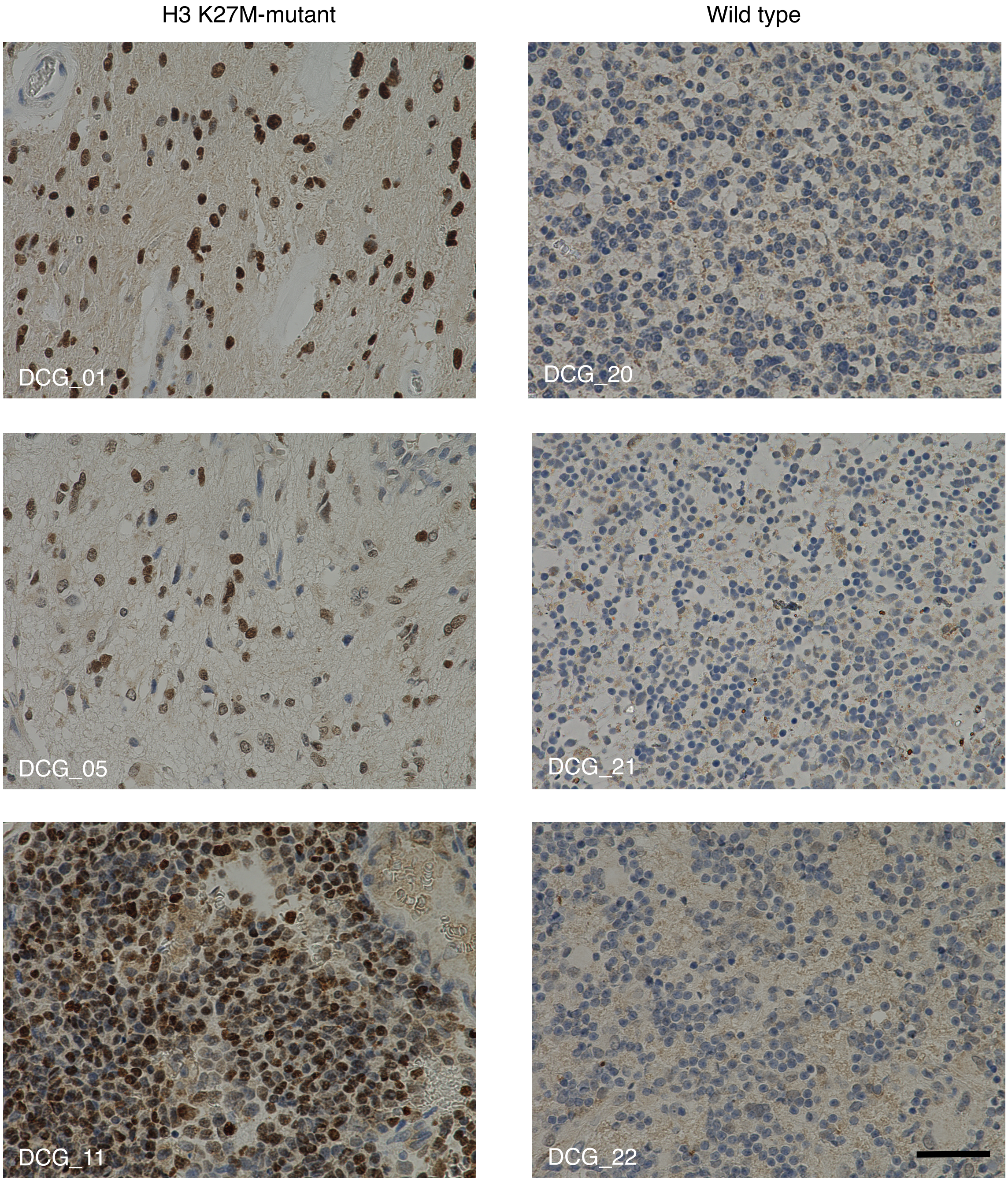

Fig. S2 Immunohistochemical staining of DCGs using anti-H3 K27M antibody. All three cases with nuclear positivity are shown on the left, and three of 24 cases without nuclear staining are shown on the right. Scale bar 50 μm.

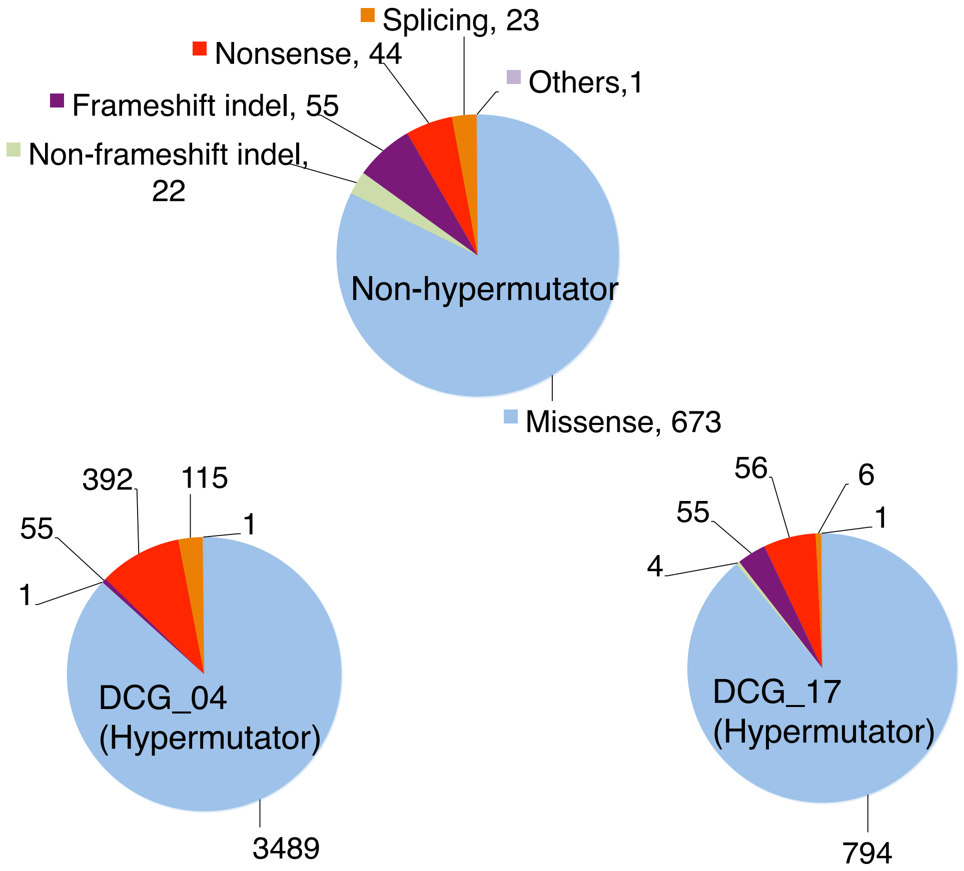

Fig. S3 Mutation load of DCGs. Pie charts showing the number of different types of somatic mutations in 15 non-hypermutator samples and in two hypermutator samples (DCG_04 and DCG_17).

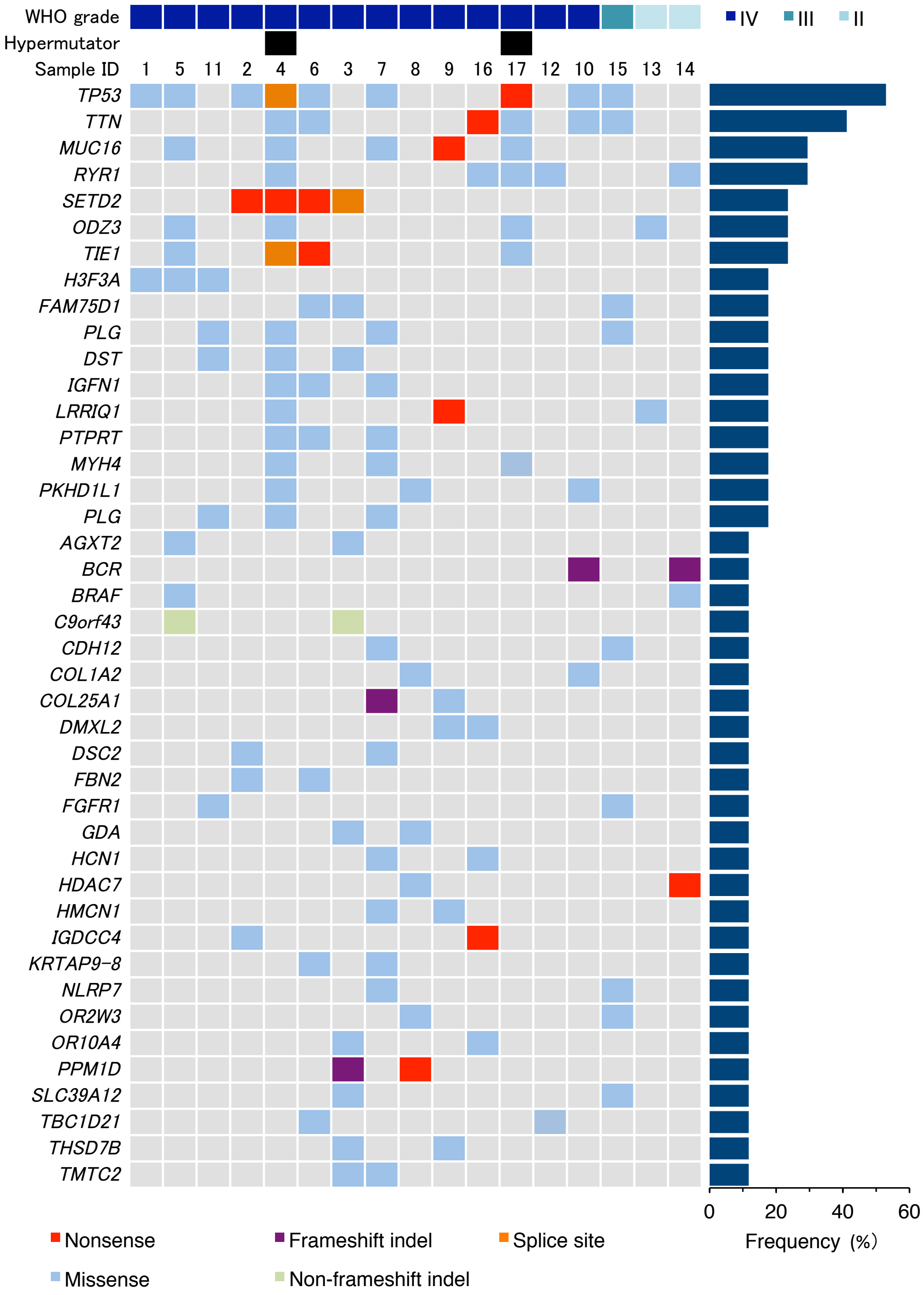

Fig. S4 WES results for DCGs. Genes mutated in more than two cases except for the two hypermutator cases are shown. WHO grade, hypermutator, and sample ID are indicated at the top. Types of alteration are indicated as colored boxes. Frequencies of alteration in each gene are shown on the right.

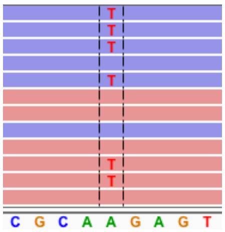

DCG_01, RNA-seq
H3F3A, Missense
Chr.1: 226252135, A to T

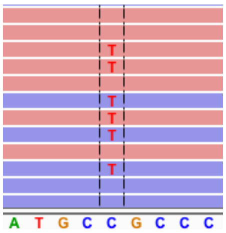

DCG_01, RNA-seq
TP53, Missense
Chr.17: 7577548, C to T

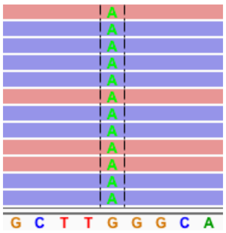

DCG_02, RNA-seq
SETD2, Nonsense
Chr.3: 47165534, G to A

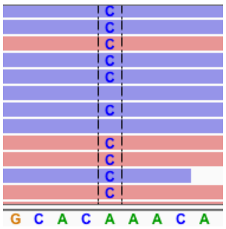

DCG_02, RNA-seq
TP53, Missense
Chr.17: 7577115, A to C

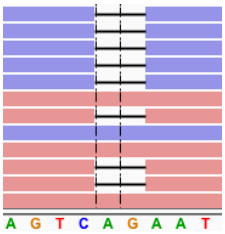

DCG_02, RNA-seq
NF1, Frameshift del
Chr.17: 29676180-81 del

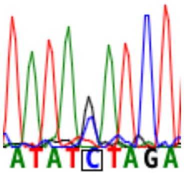

DCG_03, Sanger-seq
SETD2, Splicing
Chr.3: 47098981, C to G

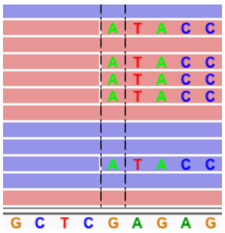

DCG_03, RNA-seq
PPM1D, Frameshift del
Chr.17: 58740467-89 del

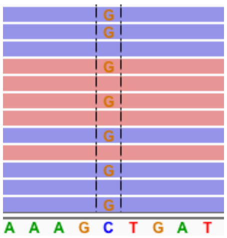

DCG_03, RNA-seq
PDGFRA, Missense
Chr.4: 55136819, C to G

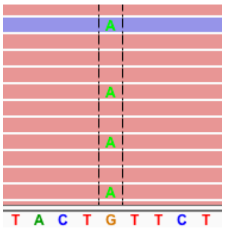

DCG_04, RNA-seq
SETD2, Nonsense
Chr.3: 47162252, G to A

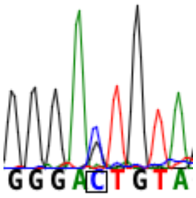

DCG_04, Sanger-seq
TP53, Splice site
Chr.17: 7579591, C to G

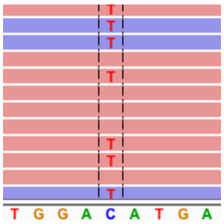

DCG_04, RNA-seq
PDGFRA, Missense
Chr.4: 55141062, C to T

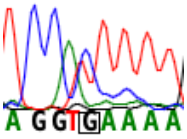

DCG_04, Sanger-seq
EGFR, Splice site
Chr.7: 55228019, G to A

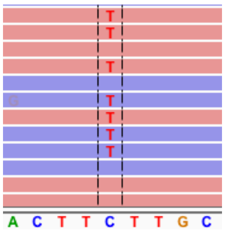

DCG_04, RNA-seq
NF1, Missense
Chr.17: 29588778, C to T

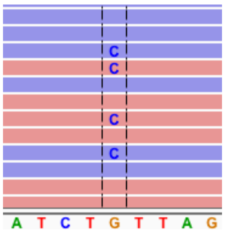

DCG_04, RNA-seq
MSH3, Missense
Chr.5: 79974908 , G to C

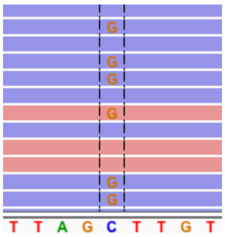

DCG_04, RNA-seq
PMS2, Missense
Chr.7: 6029468, C to G

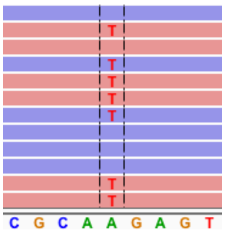

DCG_05, RNA-seq
H3F3A, Missense
Chr.1: 226252135, A to T

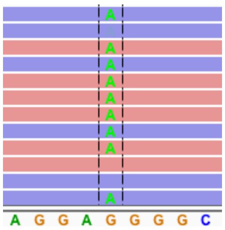

DCG_05, RNA-seq
TP53, Missense
Chr.17: 7578280, G to A

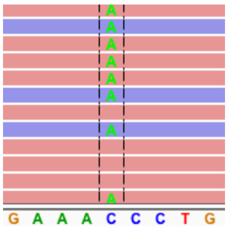

DCG_05, RNA-seq
BRAF, Missense
Chr.7: 140501299, C to A

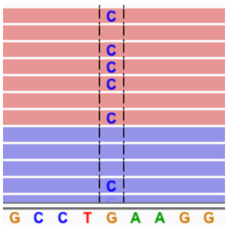

DCG_06, RNA-seq
SETD2, Nonsense
Chr.3: 47142990, G to C

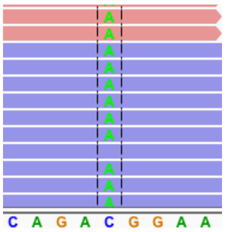

DCG_06, RNA-seq
TP53, Missense
Chr.17: 7579358, C to A

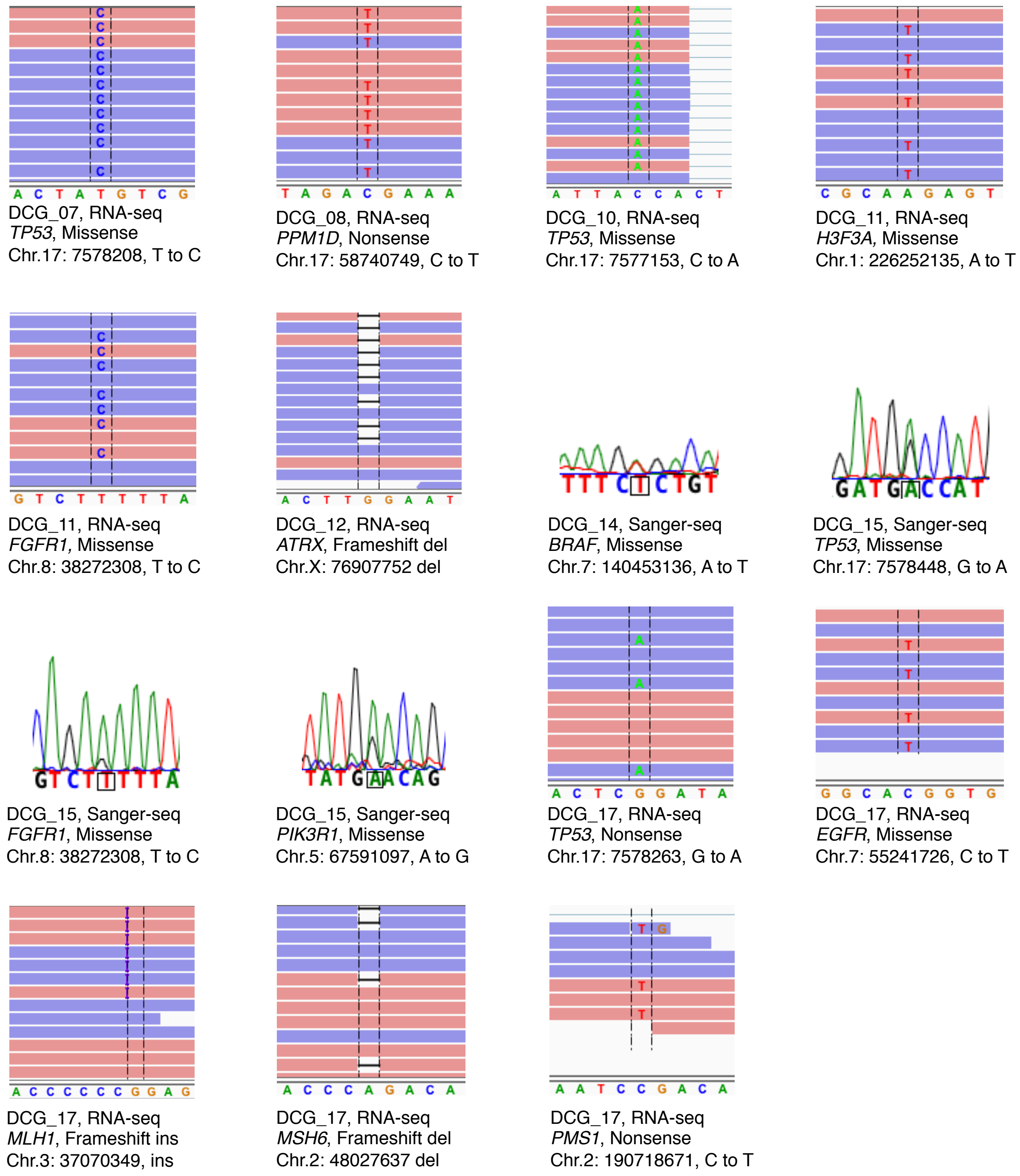

Fig. S5 Validation of mutations identified by WES. All mutations in Fig.1a were confirmed by corresponding RNA-sequencing reads or Sanger sequencing. Sample number, methods used for validation, and mutation information are shown below each graph. *RNA-seq* RNA-sequencing, *Sanger-seq* Sanger sequencing, *ins* insertion, *del* deletion.

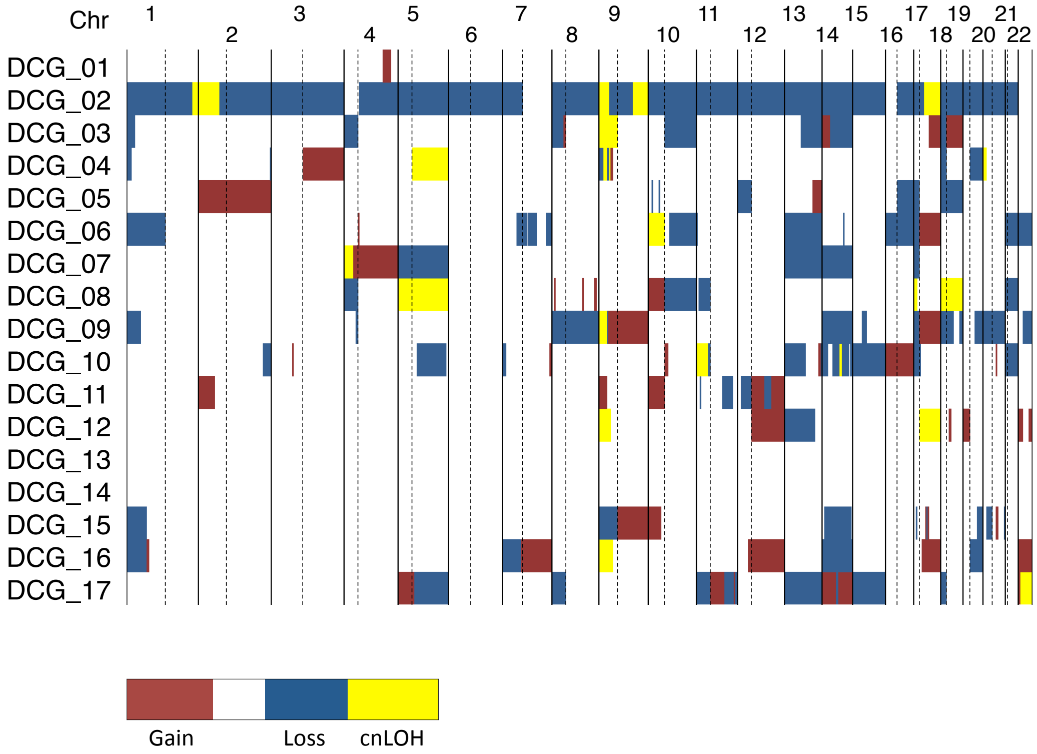

Fig. S6 Chromosome copy number alteration profiles of each sample. Chromosome copy number status is color-coded for each chromosomal site band for each patient. *Chr* chromosome, *cnLOH* copy-neutral LOH.

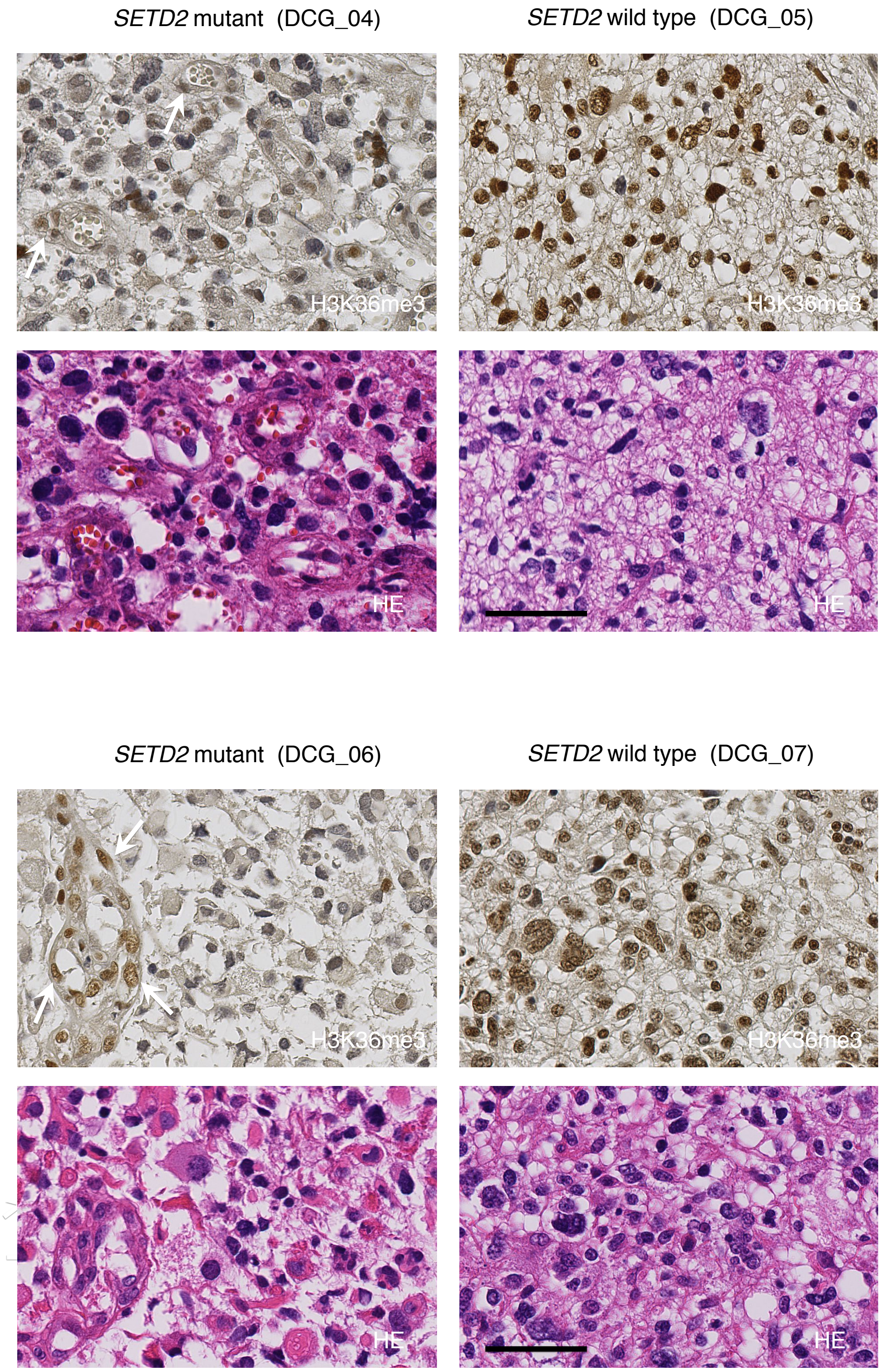

Fig. S7 Representative immunohistochemistry of H3K36 trimethylation in DCGs. Representative immunohistochemical staining of H3K36 trimethylation (H3K36me3) and hematoxylin and eosin (HE) staining of *SETD2* mutant (DCG_04 and DCG_06) and wild type (DCG_05 and DCG_07) DCGs. Nuclei of vascular endothelial cells are indicated by the white arrows as the internal positive control for H3K36me3 staining. Scale bar, 50 µm.

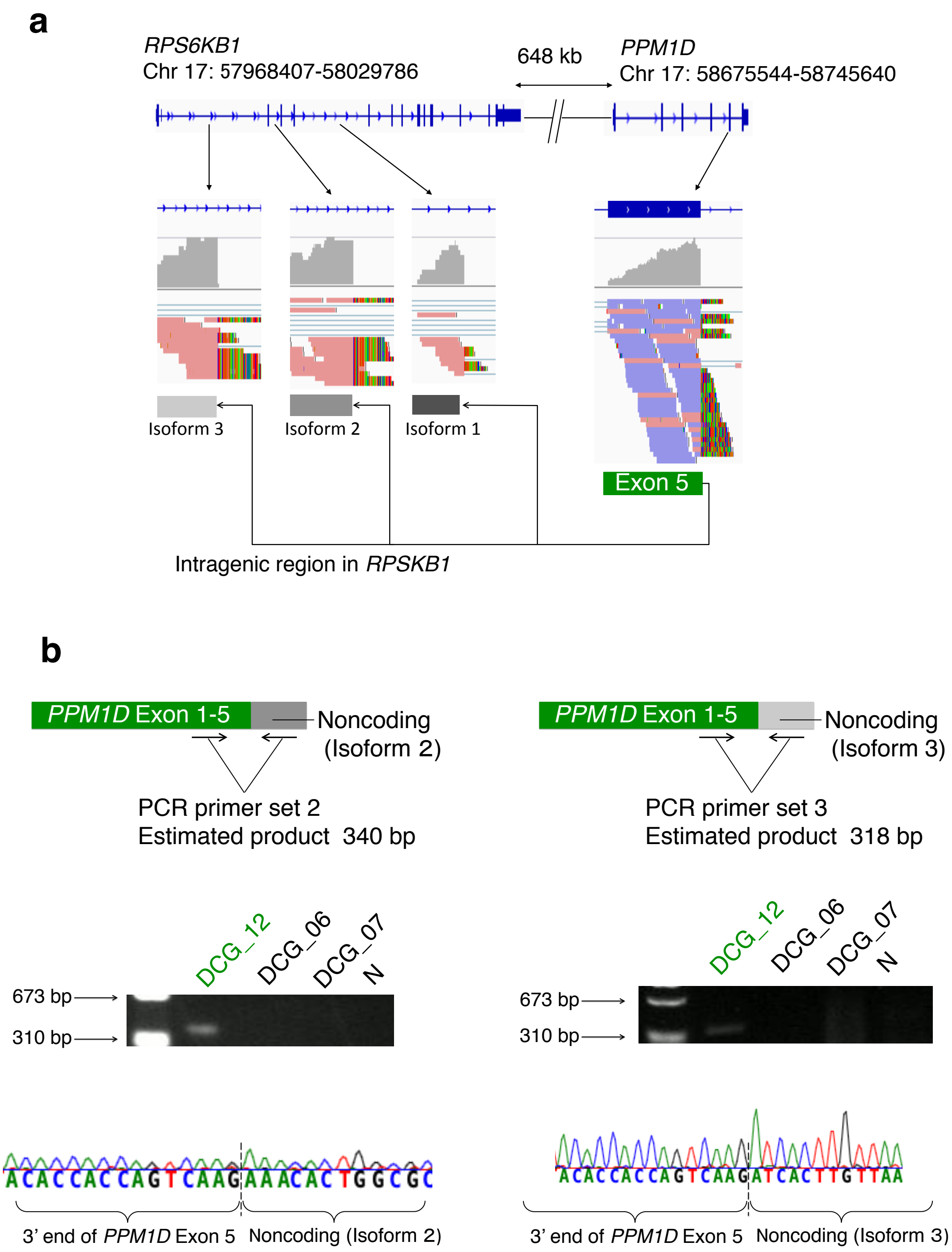

Fig. S8 Detection of *PPM1D*-noncoding (antisense *RPS6KB1* isoform 2 and 3) fusion and validation PCR. **a** Split-reads are shown aligning on *PPM1D* exon 5 and the intragenic region in *RPS6KB1*. Reads on the intragenic *RPS6KB1* region were transcribed reciprocally and included a few isoforms. *Chr* chromosome. **b** Validation PCR of the *PPM1D*-noncoding (antisense RPS6KB1 isoform 2 and 3) fusion. PCR primers were designed to specifically amplify fusion products (top). PCR bands of the estimated size of each isoform were detected in DCG_12 (middle). In a negative control lane (N), PCR product amplified without template DNA was electrophoresed. Predicted sequences were confirmed by Sanger sequencing (bottom).

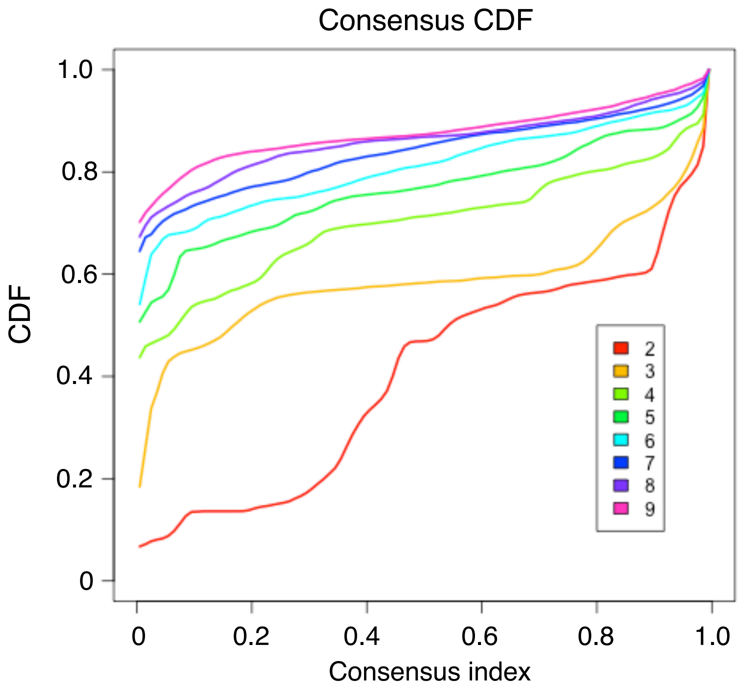

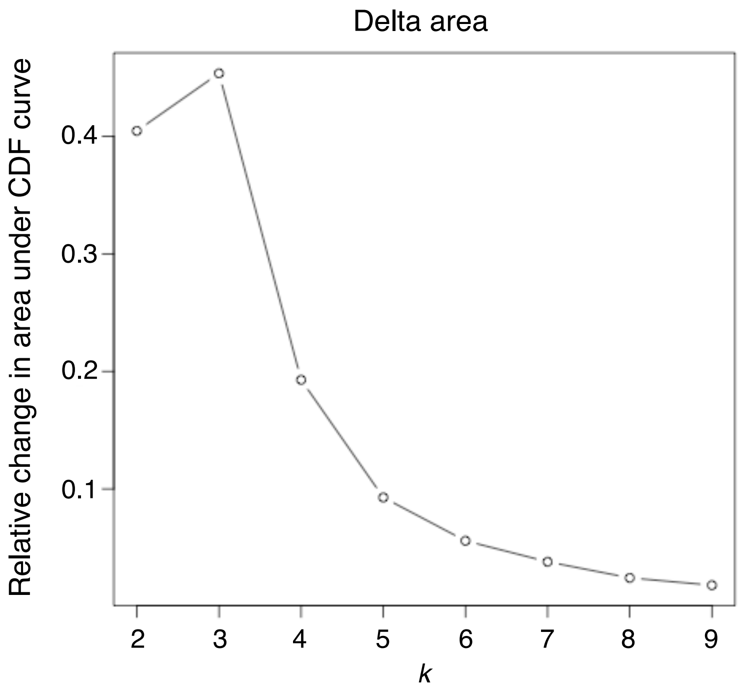

Fig. S9 Consensus *k*-means clustering of 224 gliomas. Consensus cumulative distribution function (CDF) and consecutive differences of areas under the CDF curves from *k*-means clustering for different numbers of methylation clusters are indicated. In our study, $k = 6$ was also selected as previously reported [48].

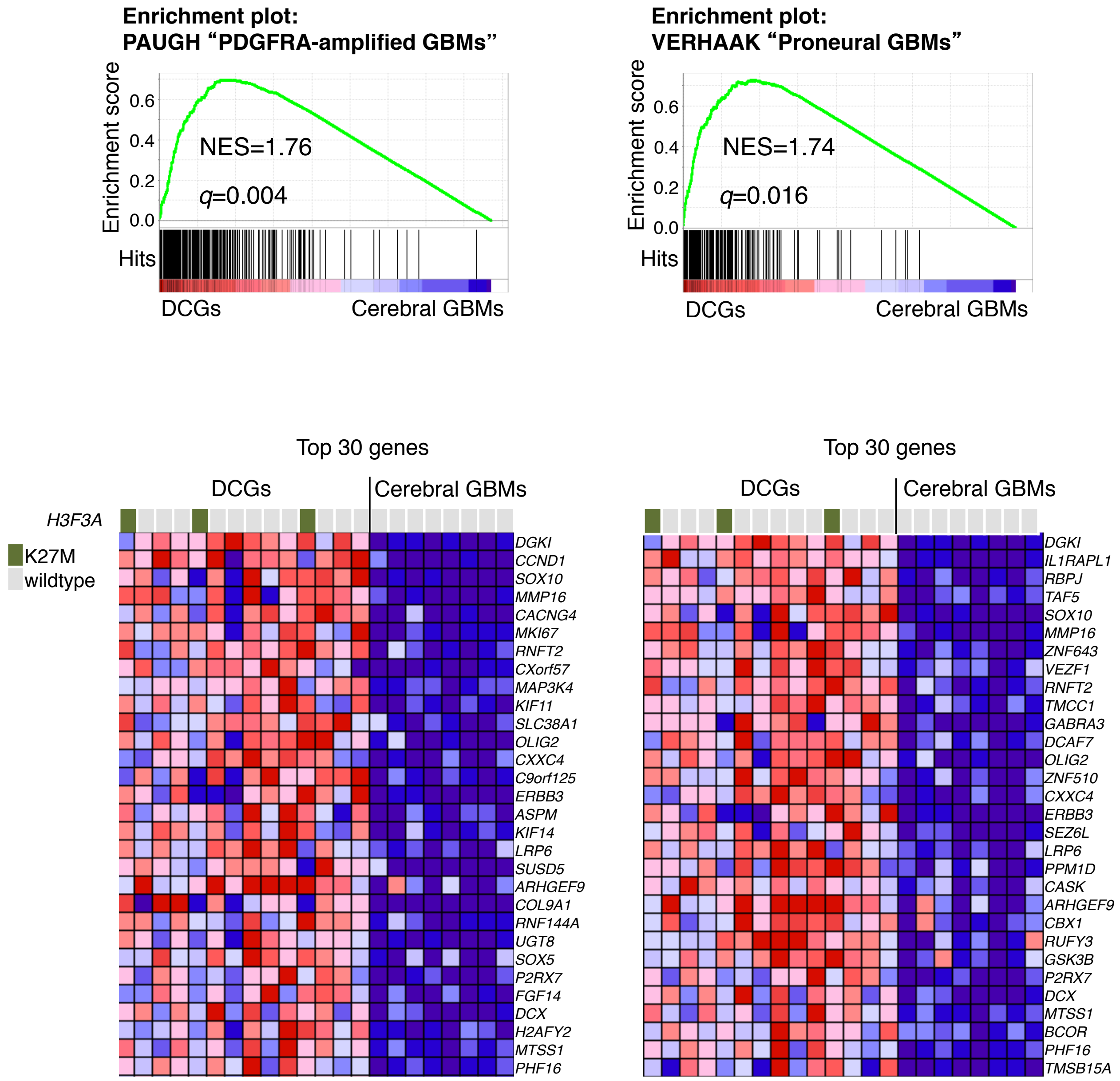

Fig. S10 Gene expression analysis of DCGs and cerebral GBMs. GSEA showed that two gene sets were up-regulated in 14 DCGs in both the "RTK I" group and the "K27" group compared with eight cerebral GBMs. One gene set was overexpressed in "PDGFRA-amplified GBMs" (left), and the other gene set was overexpressed in "Proneural GBMs" (right). The false discovery rate (*q*) and normalized enrichment score (NES) are shown on top. The top 30 significantly up-regulated genes of each gene set in DCGs are shown at the bottom. Cases with *H3F3A* K27M are indicated with green colored boxes.

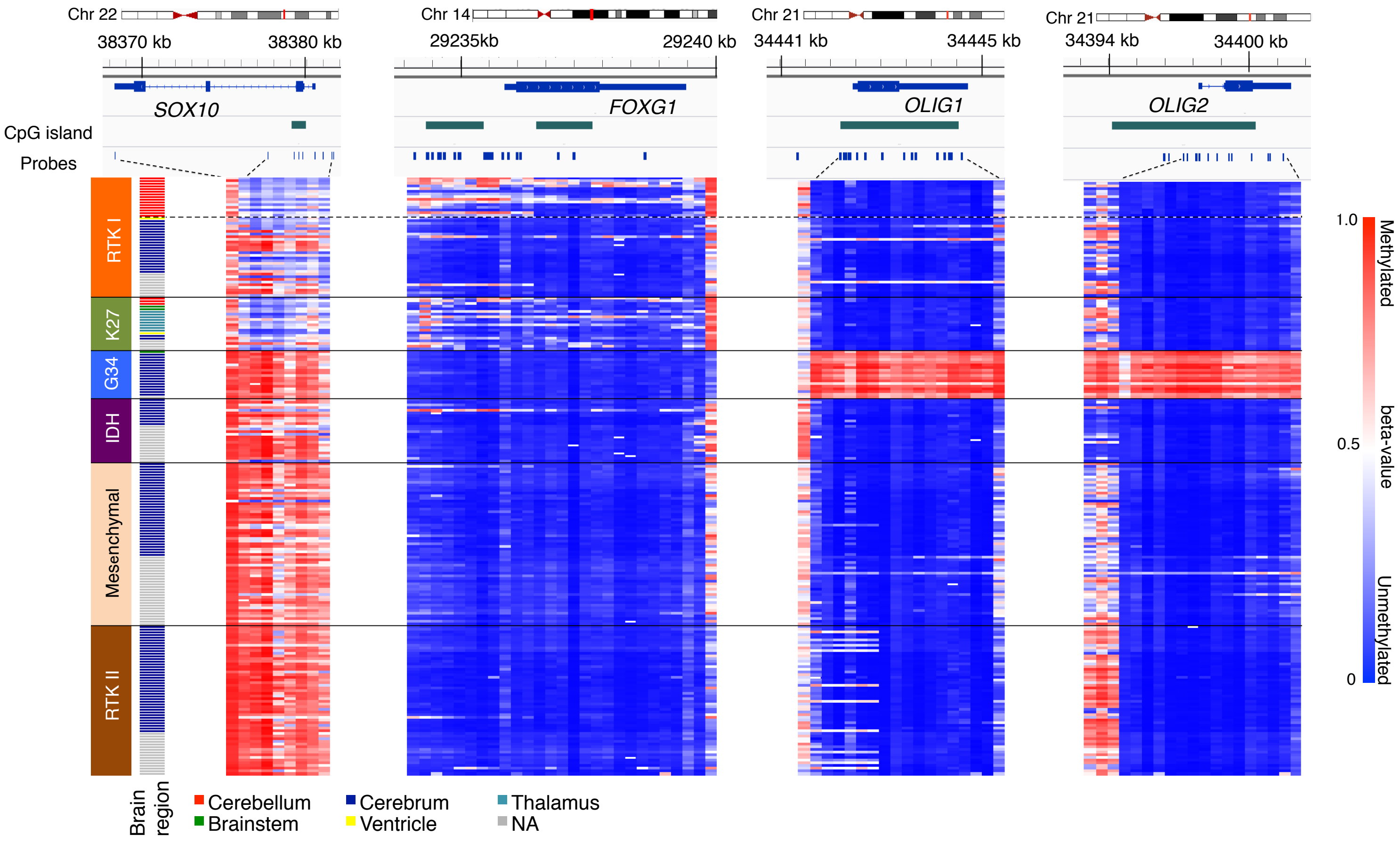

Fig. S11 Methylation level of *SOX10*, *FOXG1*, *OLIG1*, and *OLIG2* loci in 224 samples including 18 DCGs. The map of the chromosomes (Chr) of these four genes, the position of CpG islands, and the positions of the Infinium probes are shown at the top. Each row represents a sample, and each vertical bar represents an Infinium probe. Methylation clusters in this study and brain regions of each sample are shown on the left.

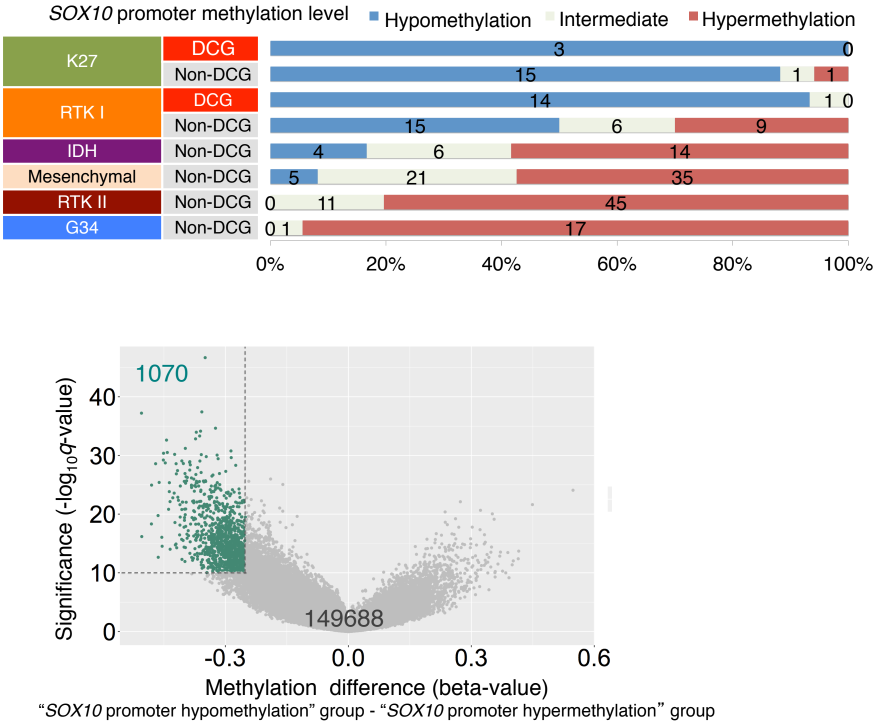

c

Rank	Motif	*p*-value	% of Targets	% of Backgrounds
1	GCGCTTTGTT	$1x10^{-26}$	54.9%	38.7%
2	CGATTCAAAG	$1x10^{-21}$	57.2%	42.3%
3	GGGTTTCATTTG	$1x10^{-19}$	0.95%	0.01%
4	ATCGATCG	$1x10^{-19}$	42.9%	29.7%
5	TATTCACG	$1x10^{-18}$	65.5%	51.9%

Fig. S12 Motifs enriched in the hypomethylated DNA region of the "*SOX10* promoter hypomethylation" group. **a** Glioma samples (n = 224) were classified into three groups depending on the *SOX10* promoter methylation level. **b** A volcano plot comparing the beta-value of each probe more than 1,500 bp from the TSS between 18 DCGs and 123 cerebral high-grade gliomas is shown. One dot represents one probe. The *q*-values that were calculated using a paired two-sided moderated Welch's *t*-test were plotted on the y-axis. Methylation differences expressed as beta-values are plotted on the x-axis. Significantly hypomethylated probes (1,070; *q*-value < 1×10^{-10} and methylation difference > 0.25) in the "*SOX10* promoter hypomethylation" group were selected. **c** Top five enriched motifs in sequences around the 1,070 hypomethylated probes of the "*SOX10* promoter hypomethylation" group are shown.
